# Adverse Health Outcomes Following Hurricane Harvey: A Comparison of Remotely‐Sensed and Self‐Reported Flood Exposure Estimates

**DOI:** 10.1029/2022GH000710

**Published:** 2023-04-21

**Authors:** Balaji Ramesh, Rashida Callender, Benjamin F. Zaitchik, Meredith Jagger, Samarth Swarup, Julia M. Gohlke

**Affiliations:** ^1^ College of Public Health The Ohio State University Columbus OH USA; ^2^ Department of Statistics Rice University Houston TX USA; ^3^ Department of Earth and Planetary Sciences Johns Hopkins University Baltimore MD USA; ^4^ Independent Consultant Austin TX USA; ^5^ Biocomplexity Institute University of Virginia Charlottesville VA USA; ^6^ Department of Population Health Sciences Virginia Tech Blacksburg VA USA; ^7^ Environmental Defense Fund Washington DC USA

**Keywords:** flood exposure assessment, remote sensing, disaster recovery, self‐reported versus remote‐sensed flood exposure, adverse health outcomes, Hurricane Harvey

## Abstract

Remotely sensed inundation may help to rapidly identify areas in need of aid during and following floods. Here we evaluate the utility of daily remotely sensed flood inundation measures and estimate their congruence with self‐reported home flooding and health outcomes collected via the Texas Flood Registry (TFR) following Hurricane Harvey. Daily flood inundation for 14 days following the landfall of Hurricane Harvey was acquired from FloodScan. Flood exposure, including number of days flooded and flood depth was assigned to geocoded home addresses of TFR respondents (*N* = 18,920 from 47 counties). Discordance between remotely‐sensed flooding and self‐reported home flooding was measured. Modified Poisson regression models were implemented to estimate risk ratios (RRs) for adverse health outcomes following flood exposure, controlling for potential individual level confounders. Respondents whose home was in a flooded area based on remotely‐sensed data were more likely to report injury (RR = 1.5, 95% CI: 1.27–1.77), concentration problems (1.36, 95% CI: 1.25–1.49), skin rash (1.31, 95% CI: 1.15–1.48), illness (1.29, 95% CI: 1.17–1.43), headaches (1.09, 95% CI: 1.03–1.16), and runny nose (1.07, 95% CI: 1.03–1.11) compared to respondents whose home was not flooded. Effect sizes were larger when exposure was estimated using respondent‐reported home flooding. Near‐real time remote sensing‐based flood products may help to prioritize areas in need of assistance when on the ground measures are not accessible.

## Key Points

- Remote sensing identified exposure to floods was associated with self‐reported flood related adverse health symptoms, illness, and injury
- Self‐reported illness and injury increased with increase in proximity to the flood waters
- The association estimated using self‐reported exposure to flood were stronger than those estimated using remote sensing

## 1 Introduction

Flooding causes acute injuries and drowning deaths and has been shown to be associated with increases in acute respiratory infections, diarrhea, skin infections, PTSD, anxiety, depression, and pregnancy complications for longer periods of time following flooding events (Ahern et al., 2005; Bevilacqua et al., 2020; Fitzpatrick, 2021; Grineski et al., 2020; Kraay et al., 2020; D. D. Saulnier et al., 2017; D. Saulnier et al., 2018; Tempark et al., 2013; Weinberger et al., 2021; Xiao et al., 2019). Furthermore, the depth of flood waters and the duration of flooding is positively associated with adverse health outcomes (Guo et al., 2020; Reacher et al., 2004). While emergency response activities include active on the ground and airplane monitoring of flooding, some areas become inaccessible and weather conditions often present dangers to emergency response personnel. Remotely sensed, real‐time identification of flood extent and depth of flood waters may aid in identifying areas most in need of aid; however, the utility of these products has not been systematically evaluated.

Hurricane Harvey, a category 4 hurricane, brought disastrous flooding to the southeast part of Texas after its landfall on 26 August 2017 (Blake & Zelinsky, 2018). Approximately 1 million hectares of land area was inundated after the landfall, and the inundation persisted for more than a week in some regions (Brakenridge & Kettner, 2017; USGS, 2020). Thousands of buildings were flooded, and electricity and utilities remained offline for weeks in some regions due to the impact of the flooding (Blake & Zelinsky, 2018). People affected by flooding either directly or indirectly had increased risk for flood related health outcomes (Grineski et al., 2020; Miranda et al., 2021; Oluyomi et al., 2021; Schwartz et al., 2018a).

Remote sensing products are helpful in demarcating flooded areas to analyze the change in flood related health outcomes during and following flooding (Ramesh et al., 2022; D. Saulnier et al., 2018). Classifying the flooding status of Census tracts following Hurricane Harvey using remotely sensed data, it was found that emergency department visits related to insect bites, acute respiratory infections, pregnancy complications, and intestinal infectious diseases were increased for residents from flooded Census tracts compared to residents of non‐flooded Census tracts (Ramesh et al., 2021).

Usage of daily flood inundation extents and flood water depth modeled using satellite‐derived observations might provide new insights on the association between flooding and health outcomes, as this will enable examination of the effect of the duration of exposure to flood waters and the effect of flood water depth. We hypothesize adverse health outcomes would be heightened as duration and depth of flooding increase. Further, comparison of the association between remote sensing‐based flood exposure and health outcomes that occurred following flooding to the corresponding association between on‐ground observed flooding and health outcomes will improve our understanding of how remote sensing products can be used to estimate exposure to flood waters. Comparison between in situ and remote‐sensed estimates of air pollution and temperature and the reliability of remote sensing products for health studies is a rich area of study (Bechle et al., 2013; Becker et al., 2021; Vanos et al., 2016; White‐Newsome et al., 2013); however, similar comparisons for flood exposure are less available. In‐situ measurements of flooding are difficult to obtain due to the dynamic nature of floods and safety concerns. The current study assesses concordance between satellite‐derived estimates of flood extents and self‐reported flooding of homes collected following Hurricane Harvey by the Texas Flood Registry. Associations between presence of home flooding, water depth and duration of flooding with self‐reported health symptomology, injury, and illness are estimated.

## 2 Materials and Methods

### 2.1 Texas Flood Registry

The TFR collected information from participating Texas residents (*N* = 20, 395) about their housing and health experiences during and following Hurricane Harvey (Miranda et al., 2021). The responses were primarily collected through online survey distribution (98%). Recruitment also occurred during in‐person events utilizing paper surveys distributed with prepaid envelopes (2%). Online and paper surveys were available in English and Spanish. The survey contained questions regarding the participants' demographics, address during the impact of the hurricane, exposure to flood water during and following the hurricane, property damage, physical health, and post hurricane health symptoms and hospitalization (for the complete questionnaire, see (Miranda et al., 2021)). The April 2020 snapshot of the data set is used in this study and contains responses collected between January 2019 and April 2020. The TFR variables that were used in this study include demographics (i.e., respondents' age, gender, ethnicity, education level); geocoded home location; self‐reported level of exposure to flooding including contact with flood water, home flooding, and flooding of other homes in the respondents' block; health symptoms experienced during or following Hurricane Harvey (i.e., concentration problems, headaches, runny nose, shortness of breath, and skin rash); and hurricane related injury, illness, and hospitalizations (Table 1). All variables related to flood exposure and health outcomes (self‐reported symptoms, illness, injury, and hospitalizations) were dichotomous. The address of the respondent during the landfall of the Hurricane was used for geocoding residence location. Out of the 20,395 records, 288 records contained non‐geocodable addresses (PO boxes, blank, etc). Of the rest, 19,762 (98.3%) records were successfully geocoded using the Children's Environmental Health Initiative (CEHI)'s AutomaticGeocodingTool_V1.99. After removing records without basic individual level characteristics, the final number of records in the data set was 18,920 records.

**Table 1:** Demographic Characteristics of the Respondents by Satellite‐Derived Flood Map Classification of Respondent Home Flooded or Non‐Flooded

	Non‐flooded		Flooded		Total	
Total	16,078	(85%)	2,842	(15%)	18,920	(100%)
Age^a^						
18‐35	3,332	(21%)	394	(14%)	3,726	(20%)
36‐50	5,107	(32%)	961	(34%)	6,068	(32%)
51‐60	3,936	(24%)	743	(26%)	4,679	(25%)
>60	3,703	(23%)	744	(26%)	4,447	(24%)
Gender						
Male	3,481	(22%)	583	(21%)	4,064	(21%)
Female	12,597	(78%)	2,259	(79%)	14,856	(79%)
Race and Ethnicity						
Non‐Hispanic white	11,628	(72%)	2,280	(80%)	13,908	(74%)
Non‐Hispanic black/African American	1,370	(9%)	148	(5%)	1,518	(8%)
Hispanic	2,446	(15%)	307	(11%)	2,753	(15%)
Non‐Hispanic Asian	262	(2%)	51	(2%)	313	(2%)
Non‐Hispanic other^b^	372	(2%)	56	(2%)	428	(2%)
Education						
Bachelor's or Graduate degree	8,173	(51%)	1,436	(51%)	9,609	(51%)
Some college/an associate degree ^c^	5,556	(35%)	1,032	(36%)	6,588	(35%)
High school diploma or less ^d^	2,349	(15%)	374	(13%)	2,723	(14%)
Self‐Assessed Health Status ^e^						
Poor	631	(4%)	101	(4%)	732	(4%)
Fair	2,599	(16%)	405	(14%)	3,004	(16%)
Good	6,189	(38%)	1,104	(39%)	7,293	(39%)
Very Good	5,008	(31%)	931	(33%)	5,939	(31%)
Excellent	1,651	(10%)	301	(11%)	1,952	(10%)

*Note*. All demographics were self‐reported by the respondents. Column percentages for each demographic category are shown in parentheses.

^a^ Derived from survey date and date of birth reported by respondent.

^b^ includes Native Hawaiian/Pacific Islander category and other races.

^c^ Respondents who attended some college and/or obtained an associate degree.

^d^ Respondents who completed eighth grade and/or obtained a high school diploma.

^e^ Question from the survey: “Compared to other persons your age, would you say your health is Excellent/Very good/Good/Fair/Poor?”

### 2.2 Satellite‐Derived Daily Flood Maps

The Atmospheric and Environmental Research (AER) FloodScan product for Hurricane Harvey uses the African Risk Capacity (ARC) Flood Extent Depiction Algorithm to generate daily flood maps at a spatial resolution of 90 m from satellite observations acquired by AMSR2 (Advanced Microwave Scanning Radiometer 2) and Global Precipitation Measurement Microwave Imager (GMI) sensors (Galantowicz et al., 2018; G. John et al., 2017). Here we used the maximum daily flood extent depiction (MFED) layer of the AER FloodScan product from 27 August 2018, to 9 September 2018. The data set contained extent of flooding for each day and the cumulative maximum extent of flooding for the entire 14 days, number of days of flooding at each pixel, and inundation depth at each pixel computed as the depth from the identified surface of the flood water to the bare earth defined by Multi‐Error‐Removed Improved‐Terrain (MERIT) digital elevation model (excluding trees, buildings, and any other surface objects) (U.S. Geological Survey, 2021; Yamazaki et al., 2017). Compared to some of the other contemporary flood extent datasets, the AER flood map has the advantage of high spatial as well as temporal resolution while incorporating satellite observations of flood waters from RADAR based satellites (Brakenridge & Kettner, 2017; FEMA, 2018). However, the inundation extents are not exactly the satellite observed flooding at the 90 m resolution as they are downscaled from a coarser grid of observations using the ARC algorithm. Also, the AER flood map is a proprietary product while the others are publicly available. The geocoded home locations of TFR respondents were superimposed on the flood maps to determine flooding of respondents' home location, number of days of flooding, the depth (in feet) of inundation, and distance to flood waters (m) for homes that were not in a flooded area.

### 2.3 Statistical Analysis

This study follows a cross‐sectional study design. The dichotomous outcome variables include the five self‐reported health symptoms, injury, illness, and hospitalization. The flood exposure measures include respondent‐reported flooding (i.e., home flooded, contact with flood water, and other homes in their block flooded) and satellite‐derived estimates of flooding (i.e., flooded [binary], distance to flooding, depth of inundation, and the number of days of flooding). The other homes flooded variable was analyzed as an exposure only among respondents who reported their home was not flooded (and will be further referred to as “only other homes in the block flooded”). Duration (days), floodwater depth (ft), and distance to flooding (m) were analyzed as continuous and categorical variables (duration: 1 day, 2–3 days, >3 days, depth: <1.5 ft, 1.5–3 ft, >3 ft, and distance: >0–400 m, 400–1,100 m, >1,100 m). Modified Poisson regression models that enable robust error estimation were used to estimate risk ratio (RR) for associations between flood variables and health outcomes, while adjusting for respondents' age, gender, education, self‐health assessment, race, and ethnicity. Separate models were constructed to estimate the association between each flood exposure variable and health outcome. In models using distance to floodwaters (m), associations are reported for every 500 m increase in distance. Effect modification of the estimated RRs by demographic variables were also analyzed using interaction terms with the flood exposure variable. In the interaction models, male gender, non‐Hispanic white ethnicity/race, bachelor or higher education, and age group 18–35 were used as the reference groups. Bonferroni correction for multiple comparisons was considered for evaluation of significance of effect modification model results (alpha = 0.05/4 covariates evaluated = 0.0125). The hospitalization outcome was not analyzed in interaction models and categorical exposure models due to sparse records (<15) in some combinations of outcome, exposure, and the interaction variable. Survey records with missing values for self‐reported exposure or self‐reported outcomes were removed from the relevant exposure outcome models, therefore each exposure‐outcome model had a different final sample size (Table S1 in Supporting Information S1). For the models with binary exposures, spatial dependence of the residuals was evaluated using Moran's I statistic and when the *p*‐value for the test was significant generalized additive models (GAM) was used to re‐estimate and compare the RR while accounting for the location of the respondents using splines (Bivand et al., 2013). This study was approved by Virginia Tech IRB (#18–914) and the Rice University IRB (#IRB‐FY2020‐18).

## 3 Results

**Figure 1:**
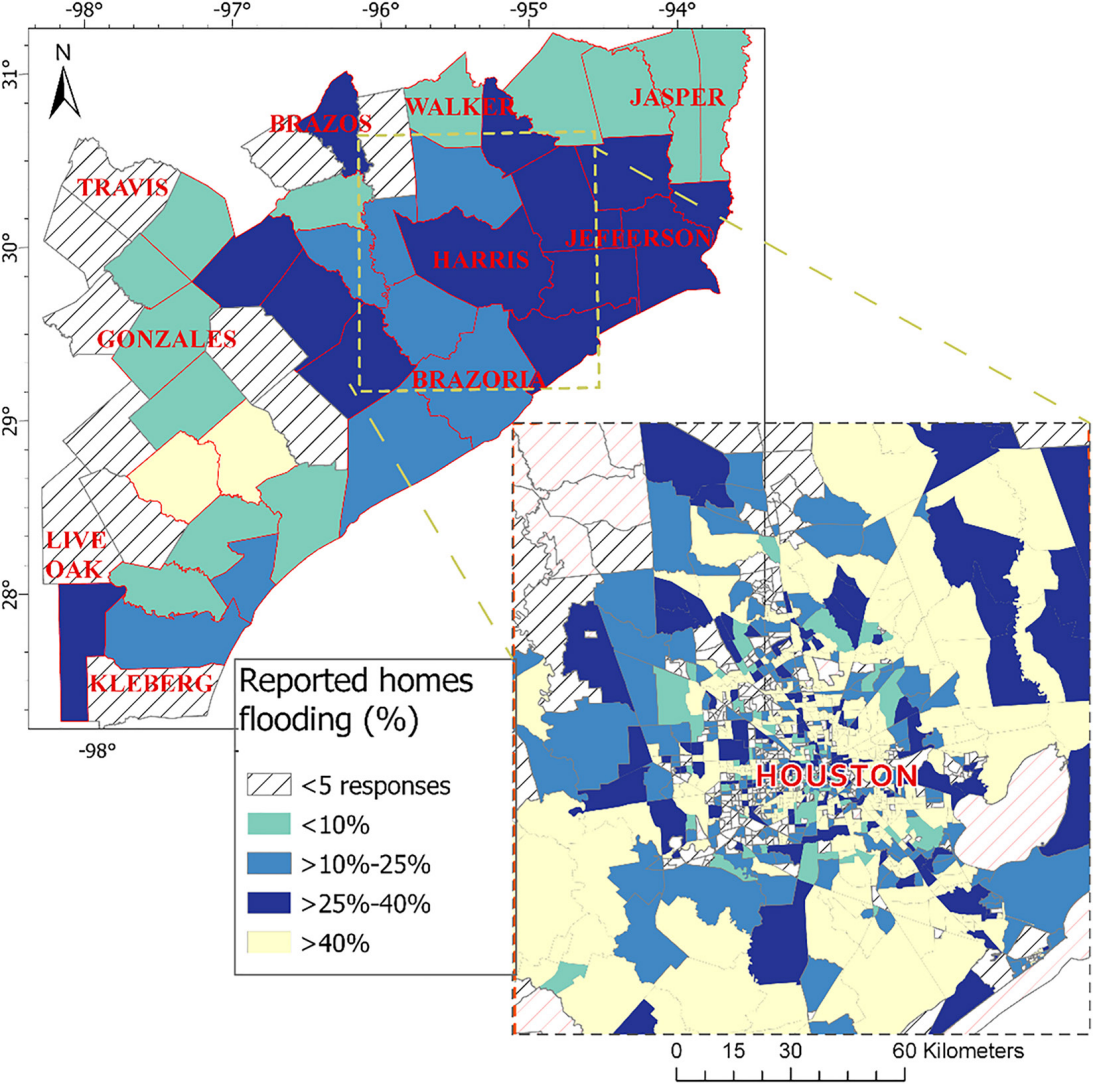
Study area as defined by counties of residence of Texas Flood Registry respondents. The map shows the percentage of respondents in each county or Census tract that reported their home to be flooded following Hurricane Harvey landfall. The main map shows the county‐level percentage of reported home flooding, and the enlarged map shows the same information at the Census tract level.

**Figure 2:**
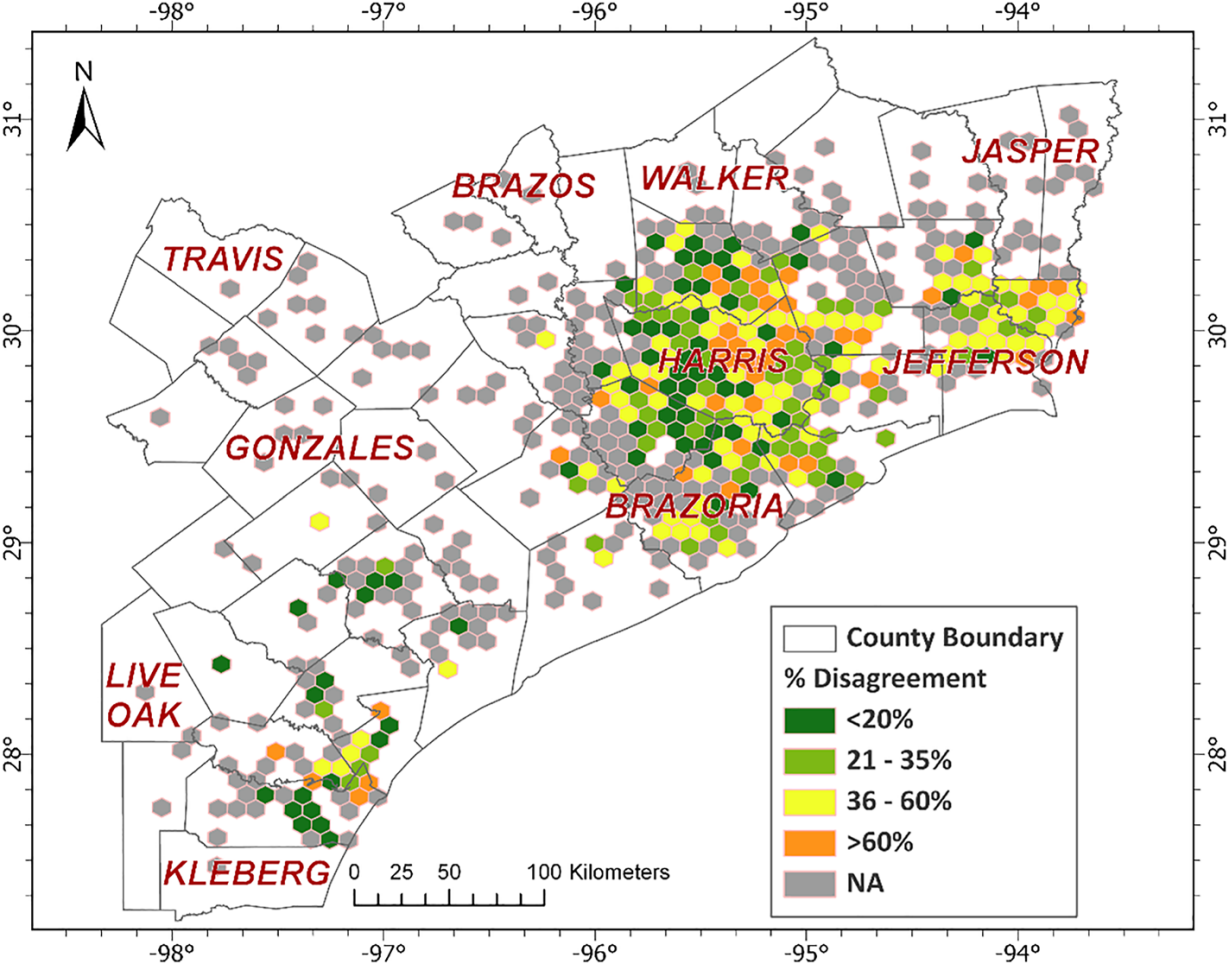
Discordance between respondent‐reported home flooding and remotely‐sensed home location flooding. Discordance determined at address level were aggregated as hexagons (area of 8,750 km2). Hexagons that contained less than five respondents are color coded with gray (NA) to protect identifiable information.

Texas Flood Registry (TFR) respondents (*N* = 18,920) belonged to 47 counties and 1,343 Census tracts (Figure 1). Most of the respondents (85%) resided in counties of the Houston Metropolitan Statistical Area (Austin, Brazoria, Chambers, Fort Bend, Galveston, Harris, Liberty, Montgomery, and Waller). Most of the respondents were female (79%), non‐Hispanic white (74%), between 36 and 60 years of age (57%) and well‐educated: 51% with a bachelor's degree or higher and 35% with an associate degree or have attended some college (Table 1). The distribution of education, gender, and self‐health assessment were similar between respondents whose home location was identified to be flooded or non‐flooded using the flood map. The proportion of white non‐Hispanic ethnicity/race among the flooded respondents (80%) was greater than that among non‐flooded respondents (72%); contrarily, a lower proportion (11% compared to 15%) of Hispanic respondents' home locations were identified to be flooded (Table 1). Out of the 18,906 TFR participants that responded to the question regarding home flooding, 44% reported their home was flooded following Hurricane Harvey. In contrast, only 15% of the participant home locations are considered to be in a flooded area based on the satellite‐derived AER FloodScan product (Table 1), resulting in a discordance between the respondent‐reported home flooding and satellite‐derived estimates of 37%. Figure 2 shows the spatial distribution of the discordance.

### 3.1 Association Between Flooding and Health Outcomes

**Figure 3:**
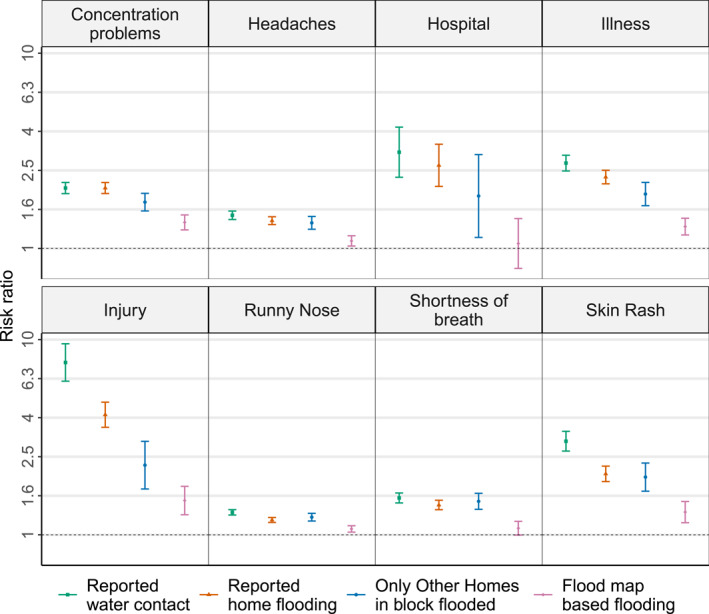
Risk ratio for association between self‐reported symptoms/injury/hospitalization and exposure to floods determined using flood map or respondents' self‐report (home flooding, other homes in the block flooded, and water contact).

Respondents whose home locations were flooded based on satellite‐derived estimates were more likely to report illness, injury, concentration problems, headaches, skin rash, shortness of breath, or runny nose compared to those whose home locations were not in flooded areas (Figure 3; Tables S2 and S3 in Supporting Information S1). The crude RRs are provided in Table S4 in Supporting Information S1. The adjusted RR for satellite‐derived flooding exposure was 1.29 (95% confidence interval [CI]: 1.17–1.43) for illness and 1.50 (95% CI: 1.27–1.77) for injury. The strength of these associations was greater when using respondent‐reported home flooding (RR = 2.32, 95% CI: 2.14–2.51 for illness and RR = 4.12, 95% CI: 3.55–4.78 for injury). Positive associations were estimated between hospitalization and self‐reported home flooding (RR = 2.67, 95% CI: 2.08–3.42) or contact with water (RR = 3.11, 95% CI: 2.32–4.18), but not when using satellite‐derived estimates of home flooding (RR = 1.06, 95% CI: 0.79–1.42). The associations between flooding and other self‐reported symptoms (i.e., concentration problems, headaches, skin rash, shortness of breath, or runny nose) were higher when using self‐reported home flooding compared to the satellite‐derived estimate of flooding (Figure 3, Table S3 in Supporting Information S1). For example, the estimated skin rash RR for self‐reported flood‐water contact was 3.02 (95% CI: 2.68–3.39), for self‐reported home flooding 2.05 (95% CI: 1.87–2.25), and for satellite‐derived flooding was 1.31 (95% CI: 1.15–1.48). Injuries showed the strongest association with self‐reported or satellite‐derived home flooding (Figure 3).

From the subset of respondents who did not report home flooding, significant positive associations were seen between flooding of other homes in their block and injury, hospitalization, and other health symptoms (Figure 3, Table S3 in Supporting Information S1). In case of illness and injury the RR estimated for this exposure (self‐report on other homes in block flooded) was 1.90 (95% CI: 1.66–2.18) and 2.27 (95% CI: 1.72–3.01) respectively, which are lower than the RRs estimated for self‐reported home flooding and greater than the RRs estimated for satellite‐derived estimates (Figure 3).

The RR estimates for the models that were identified to have residual spatial clustering were re‐estimated using GAM (Table S5 in Supporting Information S1). The statistical significance of the estimates did not change for any of the models except for model assessing the association between headache symptom and satellite derived flooding (Table S5 in Supporting Information S1).

### 3.2 Effect Modification

Effect modification (i.e., the alteration of effect of association between the flood exposure and adverse health outcomes) by age, gender, race/ethnicity, and education level were evaluated in models with self‐reported home flooding or satellite‐derived home flooding exposure variables. Detection of significant effect modification, using a conservative Bonferroni corrected alpha level of 0.0125, is presented in Table S6 in Supporting Information S1. The RRs for the association between the self‐reported home flooding and some of the self‐reported outcomes (i.e., headaches, concentration problems, runny nose, and shortness of breath) were greater among males than females (p‐values <0.015; Table S6 in Supporting Information S1). The RR for self‐reported concentration problems was greater among older age groups compared to respondents <35 years of age (p‐values <0.021; Table S6 in Supporting Information S1). No effect modification by gender or age group of the respondent was observed for the association between flood map‐identified home flooding and any of the self‐reported outcomes. Effect modification by education level was observed for both self‐reported home flooding as well as the flood map‐identified home flooding exposures in association with some of the self‐reported health outcomes; the RR was lesser among respondents without a bachelor or higher degree compared to those with bachelor or higher degree (p‐values <0.048; Table S6 in Supporting Information S1).

The effect modification by race/ethnicity was in opposite directions for self‐reported and flood map‐based home flooding in the case of the runny nose symptom. The RR was greater among black/African American respondents compared to non‐Hispanic white respondents when exposure was self‐reported home flooding and was lesser among black/African American respondents when the exposure was flood map‐based flooding (Table S6 in Supporting Information S1). Effect modification showed the RR for concentration problem and injuries to be lesser among black/African American respondents compared to non‐Hispanic white respondents for both self‐reported and flood map‐based home flooding. Risk ratio for association between concentration problem and self‐reported home flooding was also lesser among Hispanic respondents than non‐Hispanic white (*p*‐value = 0.04; Table S6 in Supporting Information S1).

### 3.3 Associations Between Health Outcomes and Flood Duration, Inundation Depth, and Distance to Flooding

#### 3.3.1 Days

For the respondents' home locations that were flooded based on satellite‐derived estimates, the days of flooding ranged between 1 and 14 days (median = 3 days). On average, for every 1 day increase in flooding, reporting of injuries increased by 10% (95% CI: 6%–14%), skin rash by 7% (95% CI: 4%–11%), concentration issues by 7% (95% CI: 4%–9%), illness by 6% (95% CI: 4%–9%), shortness of breath by 3% (95% CI: 1%–5%) and runny nose by 2% (95% CI: 1%–3%) (Table 2).

**Table 2:** Risk Ratio for the Association Between Flood Depth (ft) or Number of Days of Flooding or Distance to Flooding (per 500 m) and Self‐Reported Symptoms

	Number of days of flooding				Flood depth (ft)				Distance from flood waters (for each 500 m)			
Self‐reported outcomes	RR	95% CI		*p*‐value	RR	95% CI		*p*‐value	RR	95% CI		*p*‐value
Concentration problems	**1.07**	**1.04**	**1.09**	**<0.001**	**1.06**	**1.05**	**1.08**	**<0.001**	**0.97**	**0.95**	**0.99**	**0.001**
Headaches	**1.02**	**1.00**	**1.03**	**0.030**	1.01	1.00	1.03	0.064	0.99	0.98	1.00	0.121
Hospitalization	0.98	0.91	1.07	0.679	1.04	0.98	1.10	0.242	0.96	0.91	1.00	0.070
Illness	**1.06**	**1.04**	**1.09**	**<0.001**	**1.05**	**1.04**	**1.07**	**<0.001**	**0.97**	**0.95**	**0.99**	**0.002**
Injury	**1.10**	**1.06**	**1.14**	**<0.001**	**1.09**	**1.06**	**1.12**	**<0.001**	**0.94**	**0.90**	**0.97**	**0.001**
Runny nose	**1.02**	**1.01**	**1.03**	**<0.001**	**1.01**	**1.00**	**1.02**	**0.008**	1.00	0.99	1.00	0.213
Shortness of breath	**1.03**	**1.01**	**1.05**	**0.014**	**1.02**	**1.01**	**1.04**	**0.002**	1.00	0.99	1.01	0.712
Skin rash	**1.07**	**1.04**	**1.11**	**<0.001**	**1.05**	**1.03**	**1.08**	**<0.001**	**0.98**	**0.96**	**1.00**	**0.040**

*Note*. Statistically significant findings are in bold font.

**Figure 4:**
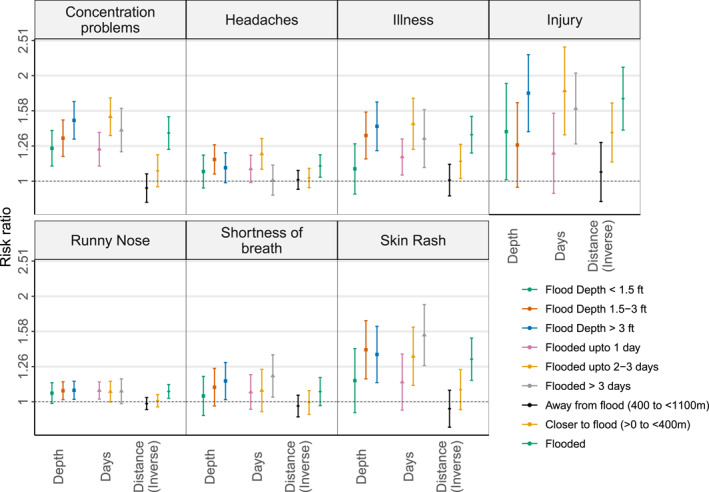
Risk ratio for association between flood depth/number of days of flooding/distance to flood waters determined using flood map and the self‐reported injury/symptoms. Reference category for flood depth and number of days is 0 (non‐flooded); and reference category for flood distance was “>1,100 m” (far away from flood). The outcome hospitalization was not analyzed due to sparse records (<15) in some combinations of the binary outcome and exposure categories.

Analyzing the number of days of inundation at respondents' home location as a categorical variable with respondents whose home location was not flooded as reference, the RR for concentration problems among respondents whose home was flooded for 2–3 days (RR = 1.53, 95% CI: 1.35–1.73) was comparatively higher than the RR among respondents whose home was flooded for 1 day (RR = 1.23, 95% CI: 1.10–1.38). A similarly high risk‐ratio for injury and illness was observed among respondents whose home was flooded for 2–3 days compared to those whose home location was flooded for 1 day (Figure 4, Table S7 in Supporting Information S1). The RR for skin rash for number of day(s) of inundation of 1, 2–3, and >3 was 1.14 (95% CI: 0.95–1.37), 1.35 (95% CI: 1.11–1.63), and 1.55 (95% CI: 1.27–1.89), respectively. The RR for headaches was elevated only among respondents whose home was flooded for 2–3 days (RR = 1.20, 95% CI: 1.08–1.32).

#### 3.3.2 Depth

The depth of inundation at the respondent home location, estimated using the AER FloodScan product varied from less than 1–32 ft (extreme values might be due to inherent errors of the flood area mapping algorithm or measurements at high‐raised houses near waterbodies) with mean of 0.5 ft (SD = 1.53 ft). For every one‐foot increase in estimated depth of floodwaters, the number of respondents who reported injury increased by 9% (95% CI: 6%–12%) on average. Similar associations were found for all self‐reported symptoms except headache (Table 2).

The data were also analyzed categorically. Compared to respondents whose home was not flooded, respondents whose home location was inundated 1.5 ft, or more were more likely to report runny nose, illness, and skin rash (Figure 4, Table S8 in Supporting Information S1). Shortness of breath was elevated only among respondents whose home location was flooded by more than 3 ft (RR = 1.15, 95% CI: 1.01–1.29) (Figure 4). For concentration problems, the RR related to inundation less than 1.5 ft, 1.5–3 ft, and greater than 3 ft were 1.24 (95% CI: 1.10–1.39), 1.33 (95% CI: 1.18–1.49), and 1.49 (95% CI: 1.32–1.68), respectively.

#### 3.3.3 Flooding Distance

Based on the AER flood map, the distance between flood waters and respondent's home locations ranged from 0 (flooded) to 8,795 m with mean of 917 m (SD = 1,122 m). For every 500 m increase in distance between the respondents' home location and the flood water, the reporting of injury, illness, and concentration issues and skin rash were decreased by 6% (95% CI: 3%–10%), 3% (95% CI: 1%–5%), and 2% (95% CI: 0%–4%) respectively (Table 2).

Risk ratios were estimated by categorizing the distance to flood waters as flooded (distance = 0), closer to flood (>0 to <400 m), away from flood (400 m to <1,100 m), and far away from flood (reference category with distance >1,100 m) (Figure 4). Respondents whose home locations were closer to flood waters were more likely to report illness and injury compared to those who resided far away from the flood (Figure 4; Table S9 in Supporting Information S1). The risk for any of the outcomes among respondents whose home was moderately away from the flood (400 m to <1,100 m) was not different compared to those far away from the flood.

## 4 Conclusions

Previous analysis of the TFR data showed that residents who reported their home was flooded following Hurricane Harvey were more likely to also report increased shortness of breath, skin rash, headaches, runny nose and concentration problems (Miranda et al., 2021). The present study found that the association between self‐reported health outcomes and remote sensing‐based home flooding estimates were significant but less strong when compared to using self‐reported home flooding as the exposure estimate (Figure 3). Self‐reported headaches, runny nose, skin rash, illness, concentration problems, and injury were associated with flood map‐based home flooding.

Self‐reported hospitalization was associated with self‐reported home flooding but not with remote sensing‐based flooding in the present study. However, an increase in flood‐related emergency department visits following Hurricane Harvey among flooded Census tracts demarcated using remote sensing‐based flood maps has been shown previously using a much larger medical records data set (Ramesh et al., 2021). Previous flooding events caused by Hurricanes Sandy, Hugo, Isabel, Andrew, and Tropical Storm Imelda have also been associated with increases in emergency department visits for specific causes (Quinn et al., 1994; Ramesh et al., 2022; Sheppa et al., 1993; Smith & Graffeo, 2005; Stryckman et al., 2017; Weinberger et al., 2021).

Consistent with the current study, associations between self‐reported exposure to flood water and self‐reported skin rash and cough symptoms among survey participants from Harris County, TX following Hurricane Harvey have been reported (Oluyomi et al., 2021). Also, increases in skin infection, acute respiratory infections, and asthma‐related hospital visits among flooded areas compared to non‐flooded areas identified using remote sensing have been shown in previous studies (Ramesh et al., 2022; D. Saulnier et al., 2018). Skin rashes following flooding events might be related to contact with polluted flood water, fungal infections, infection of traumatic wounds, and insect bites (Dayrit et al., 2018; Tempark et al., 2013). Increases in insect bite‐related ED visits were observed following flooding caused by Hurricanes Harvey, Katrina, Marilyn, Opal and Tropical Storm Imelda (Centers for Disease Control and Prevention, 1996; Faul et al., 2011; Ramesh et al., 2021, 2022); and an increase in rash symptoms was observed among relief workers following Hurricane Katrina (Centers for Disease Control and Prevention, 2005). In the current study respondents that reported contact with flood water were more than two times as likely to report skin rash compared to those who did not come in contact with flood waters.

The present results are also consistent with previous reports of increased cough, shortness of breath, and runny nose symptoms following Hurricane Katrina, Rita, and Floyd (Cummings et al., 2008; Jones et al., 2013; Murray et al., 2009). Increases in respiratory health outcomes following flooding are closely associated with indoor molds or dampness (Institute of Medicine (IOM), 2004), and studies have shown increased mold growth among flood damaged homes and subsequently increased respiratory symptoms among flood‐affected home residents (Azuma et al., 2014; Jones et al., 2013; Rose & Akpinar‐Elci, 2013; Zock et al., 2002).

Previous studies have also described increased prevalence of headaches among study participants whose homes were damaged due to Hurricane Harvey (Grineski et al., 2020). Headaches are related to mental stress, and the risk for PTSD, depression and anxiety were elevated among people affected by Hurricane Harvey (Bevilacqua et al., 2020; Fitzpatrick, 2021; Schwartz et al., 2018b). Injuries may occur both during and after the flooding due to impact of flood water, collapse of structures, contact with debris, and cleanup activities, and increase in injury related emergency department visits have been reported following flooding events (C. M. Hales et al., 2020; Hendrickson et al., 1997; Miller et al., 2013). In this study, the association between flood water contact and injury was the strongest association found between the flood exposure measures and the health outcomes reported.

Use of the satellite‐derived flood estimates allowed for evaluation of flood depth and duration effects. We found increases in reported concentration problems, illness, injury, and skin rash with increased flood depth at respondents' home locations. Studies have shown similar stronger associations with increases in flood depth following flooding for gastroenteritis as well as mental health outcomes such as anxiety, depression, psychological distress and PTSD (Lamond et al., 2015; Reacher et al., 2004; Waite et al., 2017). However, previous studies have not analyzed the association between the number of days of flooding and the risk of flood related health outcomes. We were able to estimate the effect of flood duration in the current study as remote sensing‐based flood maps provides daily inundation extents.

By computing the distance of the respondent's home location from flood waters, this study found that respondents whose home was closer to flood waters but was not flooded were more likely to report illness and injury compared to those far away from the flood waters. This finding is consistent with the elevated RR observed for illness, injury, and other symptoms among respondents who reported other homes in their block to be flooded (but no flooding at their home) compared to respondents who did not report any home or block flooding (Figure 3). These results suggest indirect effects of flooding. These indirect effects include lack of transportation to medical services due to inundated roads, interruption in essential services like electricity and water supply, or inhalation of toxic exposures from nearby flooded industrial sites, etc. In the case of Hurricane Harvey, 336,000 homes lost electricity following the landfall, and even after 3 weeks, 10 water systems and 31 waste water systems were non‐functional (Amadeo, 2019; Blake & Zelinsky, 2018). Several superfund sites in Harris County were flooded and chemical spillage from petroleum storage tanks was also reported (Karaye et al., 2019). Evidence for increased risk for respiratory symptoms among residents whose flood‐damaged home are located closer to industrial plants also exists (Azuma et al., 2014).

In the present study, older adults who reported home flooding were at higher risk for hospitalization and experiencing concentration problems after the flooding of Hurricane Harvey compared to adults younger than 35 years. Similarly, following Hurricane Sandy in 2012, older adults (65+ years) of New York City had higher rates of ED visits related to injuries, cardiovascular disease, renal disease, skin and soft tissue infections, and respiratory disease compared to other age groups (Weinberger et al., 2021). Within the present study sample, the associations between flooding and injuries or concentration problems were lower among black/African American and Hispanic respondents compared to non‐Hispanic white respondents (Table S4 in Supporting Information S1). However, this result may not be generalizable, as the TFR recruitment and sampling methods resulted in a respondent demographic distribution that does not reflect the underlying population distribution (the percentage of non‐Hispanic white, black/African American and Hispanic respondents was 74%, 8%, and 15%, respectively, while the corresponding percentages of the population in Houston based on Census data were 24%, 23%, and 45% in 2017, respectively). Additionally, the recruitment and sampling methods could have resulted in low representation of those with the most severe health impacts, as well as those with no notable impacts. However, we do note that we found similar race/ethnicity effect modification as was reported for Posttraumatic Stress Syndrome (PTSS) symptoms following Hurricane Harvey (Fitzpatrick, 2021) and ED visits for cardiovascular symptomology following Tropical Storm Imelda (Ramesh et al., 2022).

There are several reasons for the low concordance between self‐reported home flooding and satellite‐derived estimates (Figure 2). Remote sensing based inundation maps are prone to underestimate inundation in urban regions due to shadows/layover and corner reflectance caused by building and other built‐up structures, foreshortening, coverage by trees, and mixed reflectance caused by mixed features in a ground cell area (Feng et al., 2015; Kuenzer et al., 2013; Schumann et al., 2011; Schumann & Moller, 2015). Water intrusion coming from under the home or a within home infrastructure failure would not be captured by satellite. Flood maps generated using air‐borne techniques such as drones might be helpful for increasing the precision of remotely‐sensed estimates of flooding in urban areas (Backes et al., 2019; Feng et al., 2015; Kucharczyk & Hugenholtz, 2021). Recall bias in the self‐reported data set may have also contributed to the discordance seen. The agreement between AER flood map and self‐reported flooding at respondent home locations is higher in areas where respondents have reported higher depths of home flooding (Table S10 in Supporting Information S1). Remote sensing products have better sensitivity to identify flooding when the extent/area of flooding at least spans over 4 times the resolution, and greater depth of flooding would generally correspond to wider area of flooding which can be more easily captured by the satellite sensors. In this context, the self‐reported flood depth agrees with the inundation marked by AER flood map. The sensitivity of the AER map (treating self‐reported home flooding as ground truth) is 93% while the specificity is only 25% and the overall agreement between the two is 63%. Consistent with this result, a previous study comparing to flood claims datasets, suggests satellite‐derived estimates may underestimate the extent of flooding (Chen et al., 2021) and an overlay of the AER map with a FEMA map shows wide divergence (Figure S1 in Supporting Information S1). Since the self‐reported home locations are not spatially randomly distributed over the flood map extent, the concordance reported should not be considered accurate for the flood event, but rather an indication of concordance in areas that were adequately sampled by the TFR.

Remote sensing‐based flood inundation data are available within days after a flooding event, while it is difficult to implement household survey methodologies to delineate flooded areas within weeks or even months (D. Saulnier et al., 2018; Shen et al., 2019). Hence remote sensing products may be useful in post‐flood health mitigation measures for targeting communities that might experience flood‐related health outcomes. Particularly during a flood response, in the first 2 weeks, situational awareness about where there is flooding can currently be obtained safely and reliably through a few methods ‐ aerial surveillance, in situ resident self‐report (only possible if utilities are functioning), non‐systematic reports from first responders, and intentional/systematic collection of street‐level data, like Community Assessment for Public Health Emergency Response (CASPERS) (CDC, 2022), which is often not possible due to hazards. At the same time, responders depending on remotely sensed flood estimates should be aware of their uncertainties and potential to underestimate flood extent in urban environments. For this reason, systems that combine real‐time flood reporting with wide‐area remote sensing would be particularly useful. Additionally, future work could evaluate the feasibility of a real‐time data processing framework to integrate daily remotely‐sensed flood inundation extents with syndromic surveillance software (e.g., Electronic Surveillance System for the Early Notification of Community‐Based Epidemics (ESSENCE)). This integration would allow local health departments to pinpoint areas most in need of medical assistance.

## Conflict of Interest

The authors declare no conflicts of interest relevant to this study.

## Supporting information

Supporting Information S1

## Data Availability

The April 2020 snapshot data set of TFR used in this study contains personally identifiable and is not available of public but can be requested through Rice University (https://floodregistry.rice.edu/tfr). The flood map used for delineating flooding following Hurricane Harvey can be purchased from Atmospheric and Environmental Research (https://www.aer.com/weather-risk-management/floodscan-near-real-time-and-historical-flood-mapping). The authors have provided the R script used for analyses presented in this manuscript in the Supporting Information S1 and deposited it at https://doi.org/10.5281/zenodo.7767616.
