# Supplementary material for: Adverse Health Outcomes Following Hurricane Harvey: A Comparison of Remotely‐Sensed and Self‐Reported Flood Exposure Estimates
Source: Geohealth. 2023 Apr 21;7(4):e2022GH000710. doi: 10.1029/2022GH000710 (PMC10120588; doi:10.1029/2022GH000710)
Supplement: Supplementary file 1 — Supporting Information S1 [file GH2-7-e2022GH000710-s001.docx]

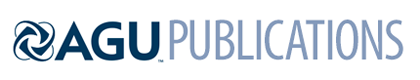


*GeoHealth*

Supporting Information for

**Adverse Health Outcomes following Hurricane Harvey: A Comparison of Remotely-sensed and Self-reported Flood Exposure Estimates**

Balaji Ramesh^1*^, Rashida Callender^2^, Benjamin F Zaitchik^3^, Meredith Jagger^4^, Samarth Swarup^5^, Julia M Gohlke^6,7*^

^1^ College of Public Health, The Ohio State University, Columbus, OH, USA, ^2^Department of Statistics, Rice University, Houston, TX, USA,^3^Department of Earth and Planetary Sciences, Johns Hopkins University, Baltimore, MD, USA,^4^Independent consultant, Austin, TX, USA,^5^Biocomplexity Institute, University of Virginia, Charlottesville, VA, USA, ^6^Department of Population Health Sciences, Virginia Tech, Blacksburg VA, USA, ^7^Environmental Defense Fund, Washington DC, USA

**Contents of this file**

Figures S1

Tables S1 to S10

R Script File

**Introduction**

Supplementary Tables and Figure present additional results from analyses, as specified in main text, along with R Script file referenced in methods section of main text.


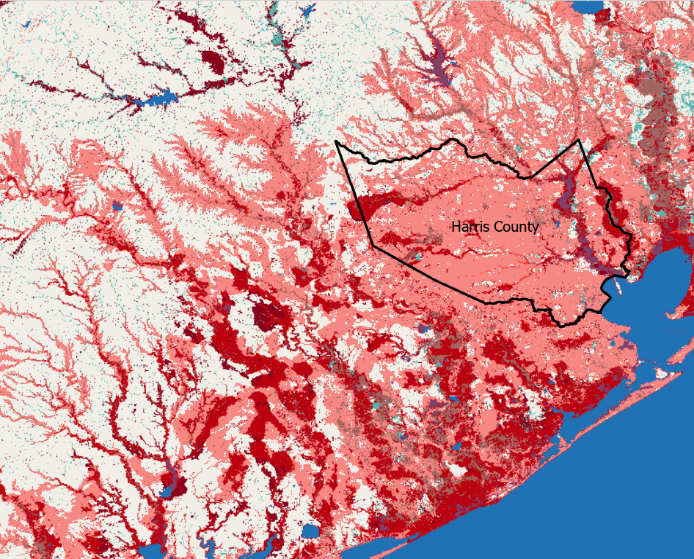

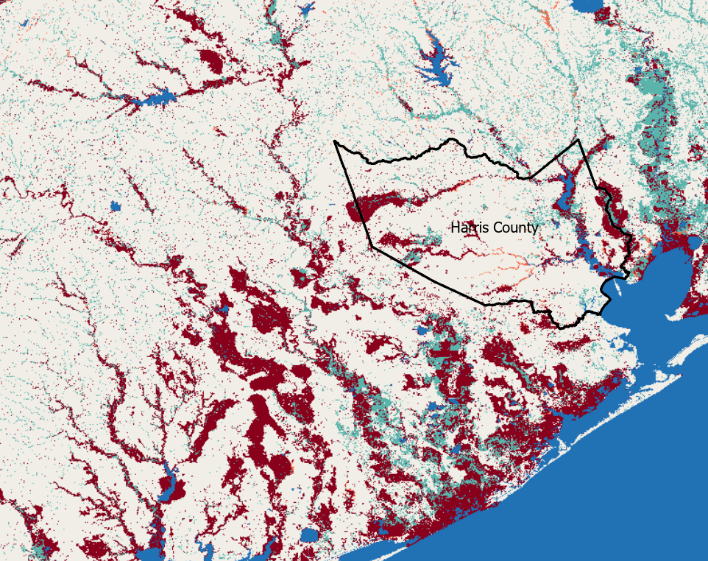


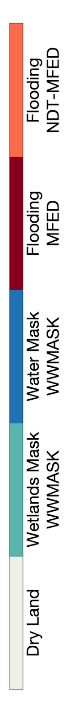


Figure S1. AER flood map (left). Flood depth from FEMA map (FEMA, 2018) categorized into inundation (> 1foot flooding) and overlaid on AER flood map (right). The light right regions on the right map are the FEMA map-based inundation.

Table S1. Sample size (n) for each model used to estimate the association (risk ratio) between the exposure to flood and the adverse health outcomes.

| **Self-reported outcomes** | **Self-reported exposure** | | | **Exposure measured using flood map and respondent home location** | | | |
| --- | --- | --- | --- | --- | --- | --- | --- |
|  | **Water contact** | **Home flooded** | **Only Other Homes in block flooded** | **Flooded (binary)** | **Flood depth** | **Number of days of flood** | **Distance to flood water** |
| Concentration problems | 18,304 | 18,299 | 8,773 | 18,303 | 18,302 | 18,302 | 18,303 |
| Headaches | 18,325 | 18,320 | 8,781 | 18,324 | 18,323 | 18,323 | 18,324 |
| Hospital | 18,913 | 18,902 | 9,022 | 18,914 | 18,913 | 18,913 | 18,914 |
| Illness | 18,804 | 18,794 | 8,974 | 18,805 | 18,804 | 18,804 | 18,805 |
| Injury | 18,860 | 18,850 | 8,996 | 18,861 | 18,860 | 18,860 | 18,861 |
| Runny nose | 18,336 | 18,333 | 8,784 | 18,334 | 18,333 | 18,333 | 18,334 |
| Shortness of breath | 18,312 | 18,307 | 8,774 | 18,310 | 18,309 | 18,309 | 18,310 |
| Skin rash | 18,282 | 18,280 | 8,766 | 18,281 | 18,280 | 18,280 | 18,281 |

Table S2. Cross tabulation for number of responses in each combination of exposure (flood map observed home location flooded) and outcome (self-reported adverse health outcomes).

| **Self-reported outcomes** | **Flood map-based home  location flooding** | |
| --- | --- | --- |
|  | **Non-Flooded** | **Flooded** |
| **Concentration problems** |  |  |
| No | 12,057 | 1,925 |
| Yes | 3,475 | 846 |
| **Headaches** |  |  |
| No | 11,197 | 1,957 |
| Yes | 4,356 | 814 |
| **Hospital** |  |  |
| No | 15,805 | 2,792 |
| Yes | 267 | 50 |
| **Illness** |  |  |
| No | 13,654 | 2,301 |
| Yes | 2,331 | 519 |
| **Injury** |  |  |
| No | 15,146 | 2,597 |
| Yes | 884 | 234 |
| **Runny nose** |  |  |
| No | 7,722 | 1,277 |
| Yes | 7,841 | 1,494 |
| **Shortness of breath** |  |  |
| No | 12,085 | 2,107 |
| Yes | 3,456 | 662 |
| **Skin rash** |  |  |
| No | 13,943 | 2,417 |
| Yes | 1,569 | 352 |

Table S3. Risk ratio (RR) for association between exposure to flood and the adverse health outcomes. Statistically significant findings are in bold font.

|  | **Water Contact (self-report)** | | | **Home flooded (self-report)** | | | **Only Other Homes in Block Flooded (self-report)** | | | **Home flooded (flood map)** | | |
| --- | --- | --- | --- | --- | --- | --- | --- | --- | --- | --- | --- | --- |
| **Self-reported outcomes** | **RR** | **95% CI** | | **RR** | **95% CI** | | **RR** | **95% CI** | | **RR** | **95% CI** | |
| Concentration problems | **2.04** | **1.91** | **2.18** | **2.04** | **1.91** | **2.18** | **1.73** | **1.56** | **1.92** | **1.36** | **1.25** | **1.49** |
| Headaches | **1.48** | **1.41** | **1.55** | **1.39** | **1.33** | **1.45** | **1.35** | **1.25** | **1.46** | **1.09** | **1.03** | **1.16** |
| Hospital | **3.11** | **2.32** | **4.18** | **2.67** | **2.08** | **3.42** | **1.86** | **1.14** | **3.03** | 1.06 | 0.79 | 1.42 |
| Illness | **2.74** | **2.49** | **3.00** | **2.32** | **2.14** | **2.51** | **1.90** | **1.66** | **2.18** | **1.29** | **1.17** | **1.43** |
| Injury | **7.63** | **6.12** | **9.52** | **4.12** | **3.55** | **4.78** | **2.27** | **1.72** | **3.01** | **1.50** | **1.27** | **1.77** |
| Runny nose | **1.30** | **1.26** | **1.35** | **1.19** | **1.16** | **1.23** | **1.23** | **1.18** | **1.29** | **1.07** | **1.03** | **1.11** |
| Shortness of breath | **1.54** | **1.46** | **1.64** | **1.42** | **1.34** | **1.50** | **1.48** | **1.35** | **1.63** | 1.08 | 1.00 | 1.17 |
| Skin rash | **3.02** | **2.68** | **3.39** | **2.05** | **1.87** | **2.25** | **1.98** | **1.67** | **2.33** | **1.31** | **1.15** | **1.48** |

Table S4. Crude risk ratio (RR) for association between exposure to flood and the adverse health outcomes. Statistically significant findings are in bold font.

|  | **Water Contact (self-report)** | | | **Home flooded (self-report)** | | | **Only Other Homes in Block Flooded (self-report)** | | | **Home flooded (flood map)** | | |
| --- | --- | --- | --- | --- | --- | --- | --- | --- | --- | --- | --- | --- |
| **Self-reported outcomes** | **RR** | **95% CI** | | **RR** | **95% CI** | | **RR** | **95% CI** | | **RR** | **95% CI** | |
| Concentration problems | **2.04** | **1.90** | **2.18** | **2.06** | **1.94** | **2.20** | **1.90** | **1.71** | **2.10** | **1.36** | **1.26** | **1.48** |
| Headaches | **1.54** | **1.46** | **1.62** | **1.47** | **1.40** | **1.54** | **1.57** | **1.46** | **1.69** | 1.06 | 0.99 | 1.13 |
| Hospital | **3.17** | **2.36** | **4.26** | **3.03** | **2.36** | **3.90** | **2.12** | **1.34** | **3.37** | 1.00 | 0.73 | 1.36 |
| Illness | **2.76** | **2.52** | **3.03** | **2.50** | **2.31** | **2.71** | **2.12** | **1.85** | **2.43** | **1.26** | **1.15** | **1.38** |
| Injury | **7.79** | **6.24** | **9.71** | **4.30** | **3.71** | **4.97** | **2.44** | **1.84** | **3.25** | **1.49** | **1.27** | **1.76** |
| Runny nose | **1.30** | **1.26** | **1.34** | **1.20** | **1.17** | **1.24** | **1.28** | **1.22** | **1.34** | **1.07** | **1.03** | **1.11** |
| Shortness of breath | **1.57** | **1.47** | **1.67** | **1.56** | **1.47** | **1.66** | **1.70** | **1.55** | **1.86** | 1.07 | 0.98 | 1.17 |
| Skin rash | **3.08** | **2.74** | **3.45** | **2.24** | **2.04** | **2.45** | **2.22** | **1.88** | **2.61** | **1.23** | **1.08** | **1.40** |

Table S5. Comparing risk ratio (RR) estimated using GEE models accounting for census tract level effects and GAM accounting for the spatial coordinates of the respondent’s home location during the hurricane. Results are shown only for those 22 out of 32 models where Moran’s I was significant (at alpha 0.05).

| **Outcome** | **Exposure** | **RR from GEE** | **95% CI (GEE)** | | **Moran's I** | **RR from GAM** | **95% CI (GAM)** | |
| --- | --- | --- | --- | --- | --- | --- | --- | --- |
| Illness | Only Other Homes Flooded* | 1.90 | 1.66 | 2.18 | 0.013 | 1.91 | 1.65 | 2.22 |
| Illness | Water Contact* | 2.74 | 2.49 | 3.00 | 0.001 | 2.83 | 2.57 | 3.11 |
| Injury | Water Contact* | 7.63 | 6.12 | 9.52 | 0.022 | 7.61 | 6.10 | 9.48 |
| Concentrate | Water Contact* | 2.04 | 1.91 | 2.18 | 0.000 | 2.05 | 1.91 | 2.21 |
| Headaches | Water Contact* | 1.48 | 1.41 | 1.55 | 0.010 | 1.48 | 1.39 | 1.58 |
| Runny Nose | Water Contact* | 1.30 | 1.26 | 1.35 | 0.033 | 1.30 | 1.25 | 1.36 |
| Shortness of Breath | Water Contact* | 1.54 | 1.46 | 1.64 | 0.002 | 1.56 | 1.45 | 1.67 |
| Skin Rash | Water Contact* | 3.02 | 2.68 | 3.39 | 0.020 | 3.05 | 2.71 | 3.44 |
| Illness | Home Flooded* | 2.32 | 2.14 | 2.51 | 0.011 | 2.36 | 2.18 | 2.56 |
| Concentrate | Home Flooded* | 2.04 | 1.91 | 2.18 | 0.015 | 2.04 | 1.92 | 2.18 |
| Headaches | Home Flooded* | 1.39 | 1.33 | 1.45 | 0.015 | 1.38 | 1.30 | 1.46 |
| Runny Nose | Home Flooded* | 1.19 | 1.16 | 1.23 | 0.023 | 1.19 | 1.14 | 1.24 |
| Shortness of Breath | Home Flooded* | 1.42 | 1.34 | 1.50 | 0.002 | 1.42 | 1.34 | 1.52 |
| Skin Rash | Home Flooded* | 2.05 | 1.87 | 2.25 | 0.012 | 2.05 | 1.86 | 2.26 |
| Illness | Home Flooded^+^ | 1.29 | 1.17 | 1.43 | 0.000 | 1.32 | 1.20 | 1.46 |
| Injury | Home Flooded^+^ | 1.50 | 1.27 | 1.77 | 0.000 | 1.51 | 1.30 | 1.76 |
| Concentrate | Home Flooded^+^ | 1.36 | 1.25 | 1.49 | 0.000 | 1.36 | 1.25 | 1.47 |
| Headaches | Home Flooded^+^ | 1.09 | 1.03 | 1.16 | 0.001 | 1.08 | 1.00 | 1.16 |
| Runny Nose | Home Flooded^+^ | 1.07 | 1.03 | 1.11 | 0.003 | 1.06 | 1.00 | 1.12 |
| Shortness of Breath | Home Flooded^+^ | 1.08 | 1.00 | 1.17 | 0.000 | 1.07 | 0.98 | 1.17 |
| Skin Rash | Home Flooded^+^ | 1.31 | 1.15 | 1.48 | 0.000 | 1.31 | 1.16 | 1.48 |

* Respondent self-reported

^+^ Flooding identified through AER flood map.

Table S6. Effect modification for association between exposure (self-reported home flooding and flood map-based flooding) and self-reported outcomes by gender, age, education, and race/ethnicity. Interactions that are significant at Bonferroni corrected confidence level (α <= 0.0125) are highlighted in bold.

|  | **Self-reported home flooding** | | | | **Flood map-based flooding** | | | |
| --- | --- | --- | --- | --- | --- | --- | --- | --- |
| **Self-reported outcomes** | **RR** | **Conf int** | | **p-value** | **RR** | **Conf int** | | **p-value** |
| **Concentration problem** |  |  |  |  |  |  |  |  |
| **Sex** |  |  |  |  |  |  |  |  |
| Female | 0.83 | 0.71 | 0.96 | 0.015 | 1.04 | 0.88 | 1.24 | 0.638 |
| **Age** |  |  |  |  |  |  |  |  |
| 36-50 | 1.21 | 1.03 | 1.42 | 0.021 | 1.21 | 0.96 | 1.52 | 0.105 |
| 51-60 | **1.26** | **1.06** | **1.49** | **0.007** | 1.20 | 0.95 | 1.50 | 0.121 |
| >60 | 1.43 | 1.19 | 1.71 | 0 | 1.23 | 0.96 | 1.58 | 0.109 |
| **Education** |  |  |  |  |  |  |  |  |
| Some college / an associate degree | 0.68 | 0.61 | 0.77 | 0 | **0.75** | **0.65** | **0.86** | **0** |
| High school diploma or less | 0.65 | 0.54 | 0.77 | 0 | 0.84 | 0.68 | 1.03 | 0.1 |
| **Race/Ethnicity** |  |  |  |  |  |  |  |  |
| Non-Hispanic black | **0.74** | **0.60** | **0.91** | **0.004** | 0.73 | 0.54 | 0.99 | 0.042 |
| Hispanic | **0.76** | **0.65** | **0.89** | **0.001** | 0.93 | 0.77 | 1.13 | 0.487 |
| Non-Hispanic Asian | 0.79 | 0.60 | 1.03 | 0.084 | 1.20 | 0.92 | 1.57 | 0.188 |
| **Headaches** |  |  |  |  |  |  |  |  |
| **Sex** |  |  |  |  |  |  |  |  |
| Female | **0.69** | **0.60** | **0.80** | **0** | 0.90 | 0.74 | 1.10 | 0.309 |
| **Age** |  |  |  |  |  |  |  |  |
| 36-50 | 1.07 | 0.95 | 1.21 | 0.237 | 1.14 | 0.97 | 1.33 | 0.105 |
| 51-60 | 1.07 | 0.94 | 1.22 | 0.28 | 1.04 | 0.86 | 1.27 | 0.687 |
| >60 | 1.10 | 0.95 | 1.29 | 0.213 | 1.00 | 0.79 | 1.26 | 0.981 |
| **Education** |  |  |  |  |  |  |  |  |
| Some college / an associate degree | 0.92 | 0.83 | 1.01 | 0.077 | 0.94 | 0.82 | 1.07 | 0.358 |
| High school diploma or less | 0.87 | 0.77 | 1.00 | 0.045 | 0.94 | 0.78 | 1.12 | 0.454 |
| **Race/Ethnicity** |  |  |  |  |  |  |  |  |
| Non-Hispanic black | 1.10 | 0.94 | 1.29 | 0.239 | 0.78 | 0.60 | 1.02 | 0.064 |
| Hispanic | 1.03 | 0.91 | 1.16 | 0.647 | 1.12 | 0.95 | 1.32 | 0.178 |
| Non-Hispanic Asian | 0.92 | 0.75 | 1.13 | 0.421 | 1.15 | 0.91 | 1.47 | 0.242 |
| **Illness** |  |  |  |  |  |  |  |  |
| **Sex** |  |  |  |  |  |  |  |  |
| Female | 0.94 | 0.76 | 1.15 | 0.535 | 0.99 | 0.78 | 1.24 | 0.898 |
| **Age** |  |  |  |  |  |  |  |  |
| 36-50 | 1.06 | 0.85 | 1.32 | 0.614 | 1.02 | 0.77 | 1.35 | 0.869 |
| 51-60 | 1.25 | 0.99 | 1.59 | 0.065 | 1.17 | 0.87 | 1.57 | 0.312 |
| >60 | 1.28 | 0.99 | 1.65 | 0.061 | 1.03 | 0.75 | 1.42 | 0.865 |
| **Education** |  |  |  |  |  |  |  |  |
| Some college / an associate degree | 0.84 | 0.71 | 1.00 | 0.048 | 0.91 | 0.73 | 1.14 | 0.403 |
| High school diploma or less | 0.76 | 0.61 | 0.96 | 0.019 | 0.87 | 0.66 | 1.16 | 0.351 |
| **Race/Ethnicity** |  |  |  |  |  |  |  |  |
| Non-Hispanic black | 1.00 | 0.78 | 1.29 | 0.996 | 0.77 | 0.56 | 1.05 | 0.097 |
| Hispanic | 1.14 | 0.93 | 1.40 | 0.205 | 0.87 | 0.68 | 1.12 | 0.288 |
| Non-Hispanic Asian | 1.24 | 0.88 | 1.75 | 0.222 | 1.08 | 0.76 | 1.53 | 0.655 |
| **Injury** |  |  |  |  |  |  |  |  |
| **Sex** |  |  |  |  |  |  |  |  |
| Female | 1.07 | 0.78 | 1.46 | 0.686 | 1.05 | 0.77 | 1.45 | 0.747 |
| **Age** |  |  |  |  |  |  |  |  |
| 36-50 | 1.10 | 0.73 | 1.67 | 0.648 | 1.27 | 0.74 | 2.16 | 0.389 |
| 51-60 | 1.34 | 0.87 | 2.05 | 0.187 | 1.25 | 0.72 | 2.16 | 0.429 |
| >60 | 1.28 | 0.81 | 2.03 | 0.288 | 1.13 | 0.63 | 2.02 | 0.692 |
| **Education** |  |  |  |  |  |  |  |  |
| Some college / an associate degree | **0.60** | **0.45** | **0.81** | **0.001** | 0.56 | 0.42 | 0.75 | 0 |
| High school diploma or less | **0.55** | **0.36** | **0.84** | **0.006** | 0.69 | 0.46 | 1.03 | 0.07 |
| **Race/Ethnicity** |  |  |  |  |  |  |  |  |
| Non-Hispanic black | 0.62 | 0.40 | 0.97 | 0.035 | 0.48 | 0.24 | 0.96 | 0.038 |
| Hispanic | 1.17 | 0.74 | 1.85 | 0.502 | 0.89 | 0.58 | 1.37 | 0.601 |
| Non-Hispanic Asian | 1.45 | 0.75 | 2.80 | 0.274 | 0.99 | 0.53 | 1.88 | 0.985 |
| **Runny Nose** |  |  |  |  |  |  |  |  |
| **Sex** |  |  |  |  |  |  |  |  |
| Female | 0.86 | 0.80 | 0.93 | 0 | 0.96 | 0.84 | 1.08 | 0.48 |
| **Age** |  |  |  |  |  |  |  |  |
| 36-50 | 0.98 | 0.90 | 1.06 | 0.579 | 1.09 | 0.96 | 1.24 | 0.193 |
| 51-60 | 1.02 | 0.93 | 1.11 | 0.689 | 1.07 | 0.94 | 1.21 | 0.292 |
| >60 | 1.07 | 0.97 | 1.17 | 0.169 | 1.10 | 0.96 | 1.26 | 0.17 |
| **Education** |  |  |  |  |  |  |  |  |
| Some college / an associate degree | 0.96 | 0.91 | 1.03 | 0.243 | 0.93 | 0.84 | 1.03 | 0.147 |
| High school diploma or less | 1.01 | 0.92 | 1.11 | 0.771 | 0.96 | 0.84 | 1.09 | 0.507 |
| **Race/Ethnicity** |  |  |  |  |  |  |  |  |
| Non-Hispanic black | **1.17** | **1.05** | **1.30** | **0.006** | 0.83 | 0.70 | 0.99 | 0.04 |
| Hispanic | 0.98 | 0.90 | 1.08 | 0.701 | 1.03 | 0.92 | 1.15 | 0.631 |
| Non-Hispanic Asian | 0.96 | 0.83 | 1.10 | 0.555 | 0.96 | 0.79 | 1.17 | 0.668 |
| **Shortness of Breath** |  |  |  |  |  |  |  |  |
| **Sex** |  |  |  |  |  |  |  |  |
| Female | **0.79** | **0.68** | **0.91** | **0.002** | 0.95 | 0.77 | 1.17 | 0.605 |
| **Age** |  |  |  |  |  |  |  |  |
| 36-50 | 1.14 | 0.96 | 1.35 | 0.124 | 1.17 | 0.90 | 1.53 | 0.252 |
| 51-60 | 1.18 | 0.99 | 1.39 | 0.059 | 1.12 | 0.85 | 1.48 | 0.416 |
| >60 | 1.20 | 1.00 | 1.43 | 0.055 | 1.16 | 0.87 | 1.54 | 0.309 |
| **Education** |  |  |  |  |  |  |  |  |
| Some college / an associate degree | 0.92 | 0.81 | 1.06 | 0.248 | 0.86 | 0.71 | 1.04 | 0.115 |
| High school diploma or less | 0.89 | 0.76 | 1.04 | 0.138 | 0.89 | 0.71 | 1.13 | 0.343 |
| **Race/Ethnicity** |  |  |  |  |  |  |  |  |
| Non-Hispanic black | 1.05 | 0.88 | 1.24 | 0.592 | 0.72 | 0.54 | 0.94 | 0.016 |
| Hispanic | 1.05 | 0.90 | 1.24 | 0.536 | 0.97 | 0.77 | 1.22 | 0.779 |
| Non-Hispanic Asian | 0.95 | 0.75 | 1.20 | 0.646 | 0.91 | 0.64 | 1.30 | 0.619 |
| **Skin Rash** |  |  |  |  |  |  |  |  |
| **Sex** |  |  |  |  |  |  |  |  |
| Female | 0.87 | 0.69 | 1.08 | 0.206 | 0.96 | 0.74 | 1.26 | 0.78 |
| **Age** |  |  |  |  |  |  |  |  |
| 36-50 | 0.99 | 0.77 | 1.27 | 0.931 | 1.34 | 0.90 | 2.00 | 0.152 |
| 51-60 | 1.16 | 0.89 | 1.51 | 0.286 | 1.16 | 0.80 | 1.69 | 0.44 |
| >60 | 1.14 | 0.85 | 1.53 | 0.375 | 1.13 | 0.74 | 1.73 | 0.558 |
| **Education** |  |  |  |  |  |  |  |  |
| Some college / an associate degree | 0.92 | 0.75 | 1.12 | 0.396 | 0.79 | 0.64 | 0.98 | 0.03 |
| High school diploma or less | 0.86 | 0.65 | 1.12 | 0.261 | 0.75 | 0.53 | 1.06 | 0.1 |
| **Race/Ethnicity** |  |  |  |  |  |  |  |  |
| Non-Hispanic black | 0.99 | 0.75 | 1.30 | 0.925 | 0.81 | 0.53 | 1.23 | 0.319 |
| Hispanic | 1.17 | 0.91 | 1.49 | 0.224 | 0.88 | 0.66 | 1.16 | 0.346 |
| Non-Hispanic Asian | 0.88 | 0.61 | 1.28 | 0.502 | 1.48 | 0.98 | 2.23 | 0.063 |

Table S7. Risk ratio for association between number of days of flooding and self-reported health outcomes. The reference is non-flooded. Statistically significant findings are in bold font.

|  | **Flooded 1 day** | | | **Flooded 2-3 days** | | | **Flooded >3 days** | | |
| --- | --- | --- | --- | --- | --- | --- | --- | --- | --- |
| **Self-reported outcomes** | **RR** | **95% CI** | | **RR** | **95% CI** | | **RR** | **95% CI** | |
| Concentration problems | **1.23** | **1.10** | **1.38** | **1.53** | **1.35** | **1.73** | **1.40** | **1.21** | **1.61** |
| Headaches | 1.08 | 0.99 | 1.19 | **1.20** | **1.08** | **1.32** | 1.01 | 0.91 | 1.11 |
| Hospital | 0.89 | 0.51 | 1.52 | **1.55** | **1.04** | **2.31** | 0.77 | 0.39 | 1.54 |
| Illness | **1.17** | **1.04** | **1.32** | **1.46** | **1.23** | **1.72** | **1.32** | **1.09** | **1.60** |
| Injury | 1.20 | 0.92 | 1.56 | **1.81** | **1.35** | **2.41** | **1.61** | **1.28** | **2.03** |
| Runny nose | **1.08** | **1.02** | **1.14** | 1.07 | 1.00 | 1.14 | 1.07 | 0.99 | 1.16 |
| Shortness of breath | 1.07 | 0.95 | 1.20 | 1.08 | 0.94 | 1.24 | **1.19** | **1.03** | **1.36** |
| Skin rash | 1.14 | 0.95 | 1.37 | **1.35** | **1.11** | **1.63** | **1.55** | **1.27** | **1.89** |

Table S8. Risk ratio for association between flood depth and self-reported health outcomes. The reference is non-flooded. Statistically significant findings are in bold font.

|  | **Flood depth <1.5 ft** | | | **Flood depth 1.5-3 ft** | | | **Flood depth >3 ft** | | |
| --- | --- | --- | --- | --- | --- | --- | --- | --- | --- |
| **Self-reported outcomes** | **RR** | **95% CI** | | **RR** | **95% CI** | | **RR** | **95% CI** | |
| Concentration problems | **1.24** | **1.10** | **1.39** | **1.33** | **1.18** | **1.49** | **1.49** | **1.32** | **1.68** |
| Headaches | 1.07 | 0.96 | 1.19 | **1.15** | **1.05** | **1.27** | 1.09 | 0.99 | 1.20 |
| Hospital | 0.90 | 0.41 | 1.99 | 0.82 | 0.47 | 1.41 | 1.41 | 0.91 | 2.18 |
| Illness | 1.08 | 0.92 | 1.28 | **1.35** | **1.16** | **1.57** | **1.43** | **1.22** | **1.68** |
| Injury | **1.38** | **1.01** | **1.90** | 1.27 | 0.96 | 1.67 | **1.78** | **1.38** | **2.29** |
| Runny nose | 1.06 | 0.99 | 1.13 | **1.08** | **1.01** | **1.14** | **1.08** | **1.02** | **1.14** |
| Shortness of breath | 1.04 | 0.91 | 1.18 | 1.10 | 0.97 | 1.25 | **1.15** | **1.01** | **1.29** |
| Skin rash | 1.15 | 0.93 | 1.42 | **1.41** | **1.16** | **1.70** | **1.36** | **1.13** | **1.64** |

Table S9. Risk ratio for association between distance to flood waters and self-reported health outcomes. The reference is distance >1100 m. Statistically significant findings are in bold font.

|  | **Flooded** | | | **Distance to flood >0 to <400m** | | | **Distance to flood 400 to <1100 m** | | |
| --- | --- | --- | --- | --- | --- | --- | --- | --- | --- |
| **Self-reported outcomes** | **RR** | **95% CI** | | **RR** | **95% CI** | | **RR** | **95% CI** | |
| Concentration problems | **1.37** | **1.23** | **1.53** | 1.07 | 0.96 | 1.19 | 0.96 | 0.87 | 1.05 |
| Headaches | **1.11** | **1.03** | **1.19** | 1.02 | 0.96 | 1.09 | 1.01 | 0.95 | 1.07 |
| Hospital | 1.26 | 0.89 | 1.80 | 1.30 | 0.97 | 1.76 | 1.24 | 0.92 | 1.67 |
| Illness | **1.36** | **1.20** | **1.53** | **1.14** | **1.02** | **1.27** | 1.01 | 0.91 | 1.12 |
| Injury | **1.72** | **1.40** | **2.11** | **1.38** | **1.13** | **1.67** | 1.06 | 0.88 | 1.29 |
| Runny nose | **1.07** | **1.02** | **1.12** | 1.01 | 0.97 | 1.05 | 0.99 | 0.95 | 1.03 |
| Shortness of breath | 1.07 | 0.98 | 1.17 | 1.00 | 0.92 | 1.08 | 0.97 | 0.91 | 1.04 |
| Skin rash | **1.32** | **1.15** | **1.52** | 1.08 | 0.95 | 1.23 | 0.96 | 0.85 | 1.08 |

Table S10. Among respondents who reported home flooding, the number and percentage of respondent’s home locations identified to be flooded using AER flood map for different levels of self-reported flood depth.

| **Water depth at home location (Respondent reported)** | **Non-flooded (as per Flood map)** | **Flooded (%)** |
| --- | --- | --- |
| Less than 1 inch | 307 | 34 (10%) |
| Between 1 inch and less than 2 inches | 330 | 47 (12%) |
| Between 2 inches and less than 3 inches | 349 | 51 (13%) |
| Between 3 inches and less than 12 inches | 1522 | 333 (18%) |
| Between 12 inches and less than 14 inches | 402 | 105 (21%) |
| Between 14 inches and less than 18 inches | 344 | 98 (22%) |
| Between 18 inches and less than 24 inches | 590 | 197 (25%) |
| 2 feet (24 inches) or more | 2113 | 1187 (36%) |

Script S1.

##### R- Code used for analysis.

#libraries

library(ggpubr)

library(dplyr)

library(tidyr)

library(broom)

library(MuMIn)

library(moments)

library(geepack)

library(car)

library(openxlsx)

library(sf)

library(caret)

library(spdep)

library(pubh)

library(MASS)

#output file location

outputDir<-"K:\\Projects\\FY2020-018_HHR_Outcomes\\EoFloodHealth\\Output\\Draft\\analysisOutput02062023\\"

#functions

mQIC<-function (x){as.numeric(QIC(x)['QIC'])}

# read the TRF(Harvey) and inundation (DfO & floodScan) ------------------- N=20395

all_df<-read.csv('K:\\Projects\\FY2020-018_HHR_Outcomes\\EoFloodHealth\\Data\\Draft\\ProcessedTFR\\TFRHarveyRecordsInclPaperSurWithInundNSVI.csv',stringsAsFactors = F)

#color blid pallette

cbbPalette <- c( "#D55E00", "#0072B2","#009E73","#56B4E9", "#CC79A7", "#000000", "#E69F00","#F0E442")

#intersting columns

interstCols<-c("X","Y","SurveyResponseID","GeoID10","tractID10","DOB","Male","RaceGroup","Hispanic","EducGroup","SelfAssess",

"Contact_Water","HomeDamaged","HomeFlooded","OtherHomesFlooded","LosePower","TrashOnBlock",

"LeaveHome","LoseIncome","VehicleDamaged",

"Hospital","Illness","Injury","NoSymptoms","Concentrate","Headaches","RunnyNose","ShortBreath","SkinRash",

"DFO_R200","dfoInundDist","fScanInundDist","fScanDepth","fScanNdays","fScanMaxFloodRatio",

"reRankSVI", "reRankSVI_T1", "reRankSVI_T2", "reRankSVI_T3", "reRankSVI_T4", "WaterLevel","HomeFlooded_Days3")

#subset data that were geocodable

all_df<-subset(all_df,NGC!=1)

#subset data with geocoding-> removes -------------- N=19762

all_df<-subset(all_df,Geocoded==1)

#-----clean data-----

#filter columns which has lessthan 3000 NAs

na_cols<-sapply(all_df, function(y) sum(length(which(is.na(y)))))

nonNaCols<-row.names(data.frame(na_cols))[na_cols<3000]

#filter only interested columns

subDf<-all_df[,interstCols]

#remove records without tract id - removes 5 records ------------ N= 19757

subDf<-subDf[!is.na(subDf$GeoID10),]

#remove records out of floodscan census tracts extent - removes 7 records - N=19750

subDf<-subDf[!is.na(subDf$fScanMaxFloodRatio),]

#create age column - age during hurricane harvey (2017-08-26)

subDf$Age<-as.numeric(difftime( as.Date('2017-08-26'),as.Date(subDf$DOB,'%m/%d/%Y'), unit="weeks"))/52.25

subDf$Age<-round(subDf$Age)

#remove records without basic persnoal infor - n=19592

subDf<-subDf[complete.cases(subDf[,c("Male", "RaceGroup", "Age")]),] #HIspanic not used as it is integrated in race

#remove blank education group n=19472

subDf<-subDf[(subDf$EducGroup!='') & !is.na(subDf$EducGroup),]

#check age below 17

table(subDf[subDf$Age<18,'Age'],subDf[subDf$Age<18,'EducGroup'])

#remove age less than 17 - n=19402

subDf<-subDf[subDf$Age>17,]

#remove records without self assesment - n=18948

subDf<-subDf[!is.na(subDf$SelfAssess),]

#remove data without SVI - n=18922

subDf<-subDf[!is.na(subDf$reRankSVI),]

####-------- change variables to factors -----

subDf$Male<-factor(subDf$Male,levels=c(1,0))

levels(subDf$Male)<-c('Male','Female')

subDf = subDf %>% mutate(Male = relevel(Male, 'Female'))

subDf$Hispanic<-factor(subDf$Hispanic,levels=c(0,1))

levels(subDf$Hispanic)<-c('Non_Hispanic','Hispanic')

subDf$RaceGroup<-factor(subDf$RaceGroup,levels=c(1,2,3,4,5))

levels(subDf$RaceGroup)<-c("Non_Hispanic_White","Non_Hispanic_Black", "Hispanic", "Non_Hispanic_Asian","Non_Hispanic_Other")

subDf$EducGroup<-factor(subDf$EducGroup,levels=c("eighth","high_school", "ged", "college", "associates", "bachelors", "graduate" ))

subDf$EducGroup<-recode(subDf$EducGroup,"c('eighth', 'high_school', 'ged')='highSchoolOrLess';c('college', 'associates') = 'collegeOrAssociates';c('bachelors','graduate')='bachelorsOrHigher'")

subDf$SelfAssess<-factor(subDf$SelfAssess,labels =c('Poor','Fair','Good','Very Good','Excellent'))

#create age group column - quantile breaks -18 38 50 60 117

subDf$AgeGrp<-cut(subDf$Age,c(0,35,50,60,200),labels = c('18-35','36-50','51-60','gt60'))

#create SVI cat columns and Scale SVI to 0-100

for(i in c("reRankSVI","reRankSVI_T1","reRankSVI_T2","reRankSVI_T3","reRankSVI_T4")){

subDf[gsub('reRank',"cat_",i)]<-cut(subDf[,i],breaks=seq(0,1,.25),include.lowest=T,labels=c('lowest','midLow','midHigh','highest'))

subDf[,i]<- subDf[,i]*100

}

#inundatiaion variables reclassify: flooded, inundataion distance, number of days, depth

#flood scan boolean field

subDf$fScanFlooded<-subDf$fScanInundDist==0

#inundation distance quantile(subDf$fScanInundDist[!subDf$fScanFlooded],seq(0,1,1/3),na.rm=T) # => 80.8 404.0 1099.0 8795.3

subDf$fScanInunDisCat<-cut(subDf$fScanInundDist,breaks=c(0,80,400,1100,9000),include.lowest=T,labels=c('Flooded','lte400','lte1100','gt1100'))

#reorder

subDf$fScanInunDisCat<- factor(subDf$fScanInunDisCat,levels=c('gt1100','lte1100','lte400','Flooded'))

#inundataion days

#quantile(subDf$fScanNdays[subDf$fScanNdays!=0],seq(0,1,1/3),na.rm=T) # 0% 33.3% 66.7% 100% # 1 1 3 14

subDf$fScanNdaysCat<-cut(subDf$fScanNdays,breaks=c(-1,0,1,3,15),include.lowest=T,labels=c('noFlood','1Day','2_3Days','4_14Days'))

subDf$fScanNdaysCat[!subDf$fScanFlooded]<-'noFlood'

#inundataion depth

#chage to feet

subDf$fScanDepthFt<-subDf$fScanDepth*3.28084

#quantile(subDf$fScanDepthFt[subDf$fScanFlooded],seq(0,1,1/3),na.rm=T) #0.00 1.62 3.27 31.97

subDf$fScanDepthCat<-cut(subDf$fScanDepthFt,breaks=c(-1,0,1.5,3,33),labels=c('noFlood','lte1Dot5ft','lte3ft','gt3ft'))

subDf$fScanDepthCat[!subDf$fScanFlooded]<-'noFlood'

#exposure variables

for (i in c("HomeFlooded","Contact_Water", "HomeDamaged", "OtherHomesFlooded", "LosePower", "TrashOnBlock",

"LeaveHome","LoseIncome", "VehicleDamaged")){

subDf[,i]<-subDf[,i]==1

}

#outcome variable

#create any symptom from no symptom variable

subDf$AnySymptoms<-as.integer(subDf$NoSymptoms==0)

#create variable OnlyOtherHomesFlooded

subDf <- subDf %>% mutate(OnlyOtherHomesFlooded=case_when(

HomeFlooded==F ~ OtherHomesFlooded,

TRUE ~ NA

))

#remove duplicate responses from the same point - removes 1333 records

#subDf<-subDf[!duplicated(paste0(subDf$X,subDf$Y,sep='')),]

#change xy to projected coordinates

sfPoints<-st_transform(st_as_sf(subDf[,c('SurveyResponseID','X','Y')], coords = c("X", "Y"), crs = 4326),crs=6579)

subDf[,c('X','Y')]<-st_coordinates(sfPoints)

#summary(subDf)

#remove(all_df)

#--------------------- data cleaning ends here N=18922 without removing responses from same point---------

#xxxxxxxxxxxxxxxxxxxxxxxxxxxxxxxxxxxxxxxxxxxxxxxxxxxxxxxxxxxxxxxxxxxxxxx

## check concordance and discordance between flood map and self reported

with(subDf, table(fScanFlooded, HomeFlooded))

#accuracy using home flooded as the truth

confusionMatrix(data= as.factor(subDf$fScanFlooded), reference=as.factor(subDf$HomeFlooded))

#create agreement disagree variable

subDf$matchSelfAndFlood <- subDf$fScanFlooded == subDf$HomeFlooded

#check water level vs fscan flooded

with(subDf, table(WaterLevel,fScanFlooded ))

with(subDf, table(WaterLevel,matchSelfAndFlood ))

with(subDf, table(HomeFlooded_Days3,fScanFlooded ))

with(subDf, table(HomeFlooded_Days3,matchSelfAndFlood ))

##-------------write demographic table using base mode ------

df<-subDf[,c('fScanFlooded','cat_SVI', "matchSelfAndFlood",

"HomeFlooded",'OnlyOtherHomesFlooded',"Contact_Water", "OtherHomesFlooded",

'AgeGrp',"Male", "RaceGroup", "Hispanic", "EducGroup","SelfAssess",

"Illness", "Injury","Hospital", "Concentrate", "Headaches", "RunnyNose", "ShortBreath", "SkinRash","AnySymptoms")]

#df<-df[complete.cases(df),] #n= 19217

#demograhic tables or cross table

library(pubh)

demoCT<-cross_tab(df,fScanFlooded~., label='fscanFlooded')

print(demoCT)

write.table(demoCT, "clipboard", sep="\t")

#demograhic table by disagreement

demoCT<-cross_tab(df,matchSelfAndFlood~., label='matchSelfAndFlood')

print(demoCT)

write.table(demoCT, "clipboard", sep="\t")

summary(df)

remove(df)

#missing data summary

library(mice)

miss=md.pattern(df)

#check chi-sq and t-test for two variaables

car.data = table(df$EducGroup, df$RaceGroup)

print(car.data)

print(chisq.test(car.data))

car.data = table(df$EducGroup, df$AgeGrp)

print(car.data)

print(chisq.test(car.data))

#check a few things

#check factor references

subDf %>% select(where(is.logical)) %>% head %>% str

table(df$HomeFlooded)

# create neighbours using the X and Y coordinates provided ----

# subDf <- subDf %>% mutate(X= as.numeric(X), Y= as.numeric(Y))

subDf <- subDf %>% mutate(resp_id = row_number())

hhr_nb <- knn2nb(knearneigh(subDf[,c("X","Y")] %>% as.matrix(),longlat = F), row.names = subDf$resp_id)

##------ =========== RUN GEE MODELS ================================= ------------

##------ 1.base model without SVI interaction and controlling for self assess ------

covariates<- c('Male','AgeGrp', 'RaceGroup', 'EducGroup',"SelfAssess") #,'Hispanic'

outcomes<- c("Illness", "Injury","Hospital", "Concentrate", "Headaches", "RunnyNose", "ShortBreath", "SkinRash","AnySymptoms")

exposures<-c("OnlyOtherHomesFlooded","Contact_Water","HomeFlooded","fScanFlooded")#,"fScanInunDisCat","OtherHomesFlooded","fScanNdaysCat" ,"fScanDepthCat","fScanDepthFt","fScanInundDist","fScanNdays")

print(paste0(c('outcomes-> ',outcomes),collapse = ', '))

print(paste0(c('exposures-> ',exposures),collapse = ', '))

print(paste0(c('covariates-> ',covariates),collapse = ', '))

summary(subDf[,c(covariates,exposures,outcomes)])

outcome<-outcomes[1]

exposure<- exposures[2]

allRes<-NA

allSumm<-''

allCrosTabs<-data.frame()

for(exposure in exposures)

for(outcome in outcomes){

mformula<-paste0(outcome,' ~ ', exposure, ' + ' , paste0(covariates,collapse = ' + '))

df<-subDf[complete.cases(subDf[,c(outcome,exposure,covariates,'tractID10','resp_id', 'X','Y')]),]

df['id']<-seq(dim(df)[1])

model<-geeglm(as.formula(mformula),

id=tractID10,

data = df,

family =poisson(link='log'),

corstr = "independence")

#update correlation structure

model2 <- update(model, corstr = "exch")

model3 <- update(model, corstr = "ar1")

##select correlation structure based on smallest QIC

modelSel<-list(model,model2,model3)[which.min(unlist(lapply(list(model,model2,model3),mQIC)))][[1]]

remove(model,model2,model3)

##get summary from the selected model

qic<- QIC(modelSel)

#summary(modelSel)

print(mformula)

#hrr_nbSub <- subset(hhr_nb, subDf$resp_id %in% df$resp_id)

hrr_nbSub <- knn2nb(knearneigh(as.matrix(df[,c('X','Y')]),k=5), row.names = df$resp_id)

moran_res <- moran.test(resid(modelSel), nb2listw(hrr_nbSub, style="W", zero.policy = T))

moran_res$p.value

#broom results

resTab<-tidy(modelSel, conf.int = TRUE,exponentiate = T)

resTab[,c('estimate','p.value','conf.low','conf.high')]<-round(resTab[,c('estimate','p.value','conf.low','conf.high')],3)

resTab<-resTab[,c("term", "estimate","conf.low", "conf.high","p.value", "std.error", "statistic")]

#insert exposure,outcome and formula and Qic

resTab$qic<-qic['QIC']

resTab$outcome<-outcome

resTab$exposure<-exposure

resTab$formula<-mformula

#correlation struture

resTab$corstr<-modelSel$corstr

resTab$Nrow<-nrow(modelSel$data)

resTab$id<-as.character(modelSel$call$id)

resTab$moranIpval <- moran_res$p.value

resTab$otherCom<-""

#print(resTab)

#prepare raw summary file

sumTxt<-paste0(c(capture.output(summary(modelSel)),

'\n QIC:',capture.output(qic),

'Time:',as.character(Sys.time()),'\n'),collapse = '\n')

#replace formula with orginal formula

sumTxt<-gsub('mformula',mformula,sumTxt)

#add to all output stored df and str

if(is.na(allRes)) allRes<-resTab else allRes<-rbind(allRes,resTab)

allSumm<-paste0(allSumm,sumTxt,sep=paste0(rep('=',100),collapse=''))

#create cross table if intersection columns are factor

if(is.factor(df[,exposure]) | is.logical(df[,exposure])){

ftab<-data.frame(ftable(df[,outcome],df[,exposure],dnn=c(outcome,exposure)),stringsAsFactors = F)

ftab$formula<-mformula

ftab<-rbind(colnames(ftab),sapply(ftab,as.character))

colnames(ftab) <- as.character(seq(dim(ftab)[2]))

allCrosTabs<-rbind(allCrosTabs,ftab)

}

}

#write output files

allRes$model<-'baseModel'

write.xlsx(allRes, file=paste0(outputDir,'baseModel.xlsx'), sheetName = "Sheet1",col.names = TRUE, row.names = TRUE, append = FALSE)

write.csv(allCrosTabs,file = paste0(outputDir,'baseModel.csv'))

cat(allSumm,file = paste0(outputDir,'baseModel.txt'))

##------ 1.1 base model without SVI interaction and controlling for self assess using gam------

covariates<- c('Male','AgeGrp', 'RaceGroup', 'EducGroup',"SelfAssess") #,'Hispanic'

outcomes<- c("Illness", "Injury","Hospital", "Concentrate", "Headaches", "RunnyNose", "ShortBreath", "SkinRash","AnySymptoms")

exposures<-c("OnlyOtherHomesFlooded","Contact_Water","HomeFlooded","fScanFlooded")#,"fScanInunDisCat") #,"OtherHomesFlooded","fScanNdaysCat" ,"fScanDepthCat","fScanDepthFt","fScanInundDist","fScanNdays")

print(paste0(c('outcomes-> ',outcomes),collapse = ', '))

print(paste0(c('exposures-> ',exposures),collapse = ', '))

print(paste0(c('covariates-> ',covariates),collapse = ', '))

summary(subDf[,c(covariates,exposures,outcomes)])

outcome<-outcomes[1]

exposure<- exposures[1]

allRes<-NA

allSumm<-''

allCrosTabs<-data.frame()

library(mgcv)

for(exposure in exposures)

for(outcome in outcomes){

mformula<-paste0(outcome,' ~ ', exposure, ' + ' , paste0(covariates,collapse = ' + '),' + s(X,Y)')

df<-subDf[complete.cases(subDf[,c(outcome,exposure,covariates,'tractID10','resp_id', 'X','Y')]),]

df$X <- df$X/1000

df$Y <- df$Y/1000

df['id']<-seq(dim(df)[1])

##select correlation structure based on smallest QIC

modelSel<-gam(as.formula(mformula),

data = df,

family =poisson(link='log'))

##get summary from the selected model

qic<- AIC(modelSel)

#summary(modelSel)

print(mformula)

print(summary(modelSel))

#run morans I

#hrr_nbSub <- subset(hhr_nb, subDf$resp_id %in% df$resp_id)

hrr_nbSub <- knn2nb(knearneigh(as.matrix(df[,c('X','Y')]),k=5), row.names = df$resp_id)

moran_res <- moran.test(resid(modelSel), nb2listw(hrr_nbSub, style="W", zero.policy = T))

print(moran_res)

#broom results

# resTab<-tidy(modelSel, conf.int = TRUE,exponentiate = T)

# resTab[,c('estimate','p.value','conf.low','conf.high')]<-round(resTab[,c('estimate','p.value','conf.low','conf.high')],3)

# resTab<-resTab[,c("term", "estimate","conf.low", "conf.high","p.value", "std.error", "statistic")]

#

#insert exposure,outcome and formula and Qic

# resTab$qic<-qic['QIC']

# resTab$outcome<-outcome

# resTab$exposure<-exposure

# resTab$formula<-mformula

# #correlation struture

# resTab$corstr<-modelSel$corstr

# resTab$Nrow<-nrow(modelSel$data)

# resTab$id<-as.character(modelSel$call$id)

# resTab$otherCom<-""

# resTab$moranIpval <- moran_res$p.value

# #print(resTab)

#prepare raw summary file

sumTxt<-paste0(c(capture.output(summary(modelSel)),

'\n AIC:',capture.output(qic),

"\n MoranI: ", capture.output(moran_res),

'Time:',as.character(Sys.time()),'\n'),collapse = '\n')

#replace formula with orginal formula

sumTxt<-gsub('mformula',mformula,sumTxt)

#add to all output stored df and str

#if(is.na(allRes)) allRes<-resTab else allRes<-rbind(allRes,resTab)

allSumm<-paste0(allSumm,sumTxt,sep=paste0(rep('=',100),collapse=''))

#create cross table if intersection columns are factor

# if(is.factor(df[,exposure]) | is.logical(df[,exposure])){

# ftab<-data.frame(ftable(df[,outcome],df[,exposure],dnn=c(outcome,exposure)),stringsAsFactors = F)

# ftab$formula<-mformula

# ftab<-rbind(colnames(ftab),sapply(ftab,as.character))

# colnames(ftab) <- as.character(seq(dim(ftab)[2]))

# allCrosTabs<-rbind(allCrosTabs,ftab)

# }

}

#write output files

#allRes$model<-'baseModel_spatial'

#write.xlsx(allRes, file=paste0(outputDir,'baseModel_spatial.xlsx'), sheetName = "Sheet1",col.names = TRUE, row.names = TRUE, append = FALSE)

#write.csv(allCrosTabs,file = paste0(outputDir,'baseModel_spatial.csv'))

cat(allSumm,file = paste0(outputDir,'baseModel_spatial.txt'))

##------ 1.2. reruning base model controlling for self assess to compare homeflooded, other homes flooded, fcsn flooded with same number of records ------

covariates<- c('Male','AgeGrp', 'RaceGroup', 'EducGroup',"SelfAssess") #,'Hispanic'

outcomes<- c("Illness", "Injury","Hospital", "Concentrate", "Headaches", "RunnyNose", "ShortBreath", "SkinRash","AnySymptoms")

exposures<-c("OtherHomesFlooded","HomeFlooded","fScanFlooded")

print(paste0(c('outcomes-> ',outcomes),collapse = ', '))

print(paste0(c('exposures-> ',exposures),collapse = ', '))

print(paste0(c('covariates-> ',covariates),collapse = ', '))

summary(subDf[,c(covariates,exposures,outcomes)])

allRes<-NA

allSumm<-''

allCrosTabs<-data.frame()

library(spind)

for(exposure in exposures)

for(outcome in outcomes){

mformula<-paste0(outcome,' ~ ', exposure, ' + ' , paste0(covariates,collapse = ' + '))

df<-subDf[complete.cases(subDf[,c(outcome,exposures,covariates,'tractID10')]),]

df['id']<-seq(dim(df)[1])

df$X <- as.integer(df$X)

df$Y <- as.integer(df$Y)

modelSel <- spind::GEE(as.formula(mformula),

#id=tractID10,

data = df,

family =poisson(link='log'),

coord = df[,c("X","Y")],corstr = "exchangable",scale.fix = FALSE)

##get summary from the selected model

qic<- QIC(modelSel)

#summary(modelSel)

#broom results

resTab<-tidy(modelSel, conf.int = TRUE,exponentiate = T)

resTab[,c('estimate','p.value','conf.low','conf.high')]<-round(resTab[,c('estimate','p.value','conf.low','conf.high')],3)

resTab<-resTab[,c("term", "estimate","conf.low", "conf.high","p.value", "std.error", "statistic")]

#insert exposure,outcome and formula and Qic

resTab$qic<-qic['QIC']

resTab$outcome<-outcome

resTab$exposure<-exposure

resTab$formula<-mformula

#correlation struture

resTab$corstr<-modelSel$corstr

resTab$Nrow<-nrow(modelSel$data)

resTab$id<-as.character(modelSel$call$id)

resTab$otherCom<-""

#print(resTab)

#prepare raw summary file

sumTxt<-paste0(c(capture.output(summary(modelSel)),

'\n QIC:',capture.output(qic),

'Time:',as.character(Sys.time()),'\n'),collapse = '\n')

#replace formula with orginal formula

sumTxt<-gsub('mformula',mformula,sumTxt)

#add to all output stored df and str

if(is.na(allRes)) allRes<-resTab else allRes<-rbind(allRes,resTab)

allSumm<-paste0(allSumm,sumTxt,sep=paste0(rep('=',100),collapse=''))

print(mformula)

#create cross table if intersection columns are not factor

if(is.factor(df[,exposure]) | is.logical(df[,exposure])){

ftab<-data.frame(ftable(df[,outcome],df[,exposure],dnn=c(outcome,exposure)),stringsAsFactors = F)

ftab$formula<-mformula

ftab<-rbind(colnames(ftab),sapply(ftab,as.character))

colnames(ftab) <- as.character(seq(dim(ftab)[2]))

allCrosTabs<-rbind(allCrosTabs,ftab)

}

}

#write output files

allRes$model<-'baseModelCompRSvsReported'

write.xlsx(allRes, file=paste0(outputDir,'baseModelCompRSvsReported.xlsx'), sheetName = "Sheet1",col.names = TRUE, row.names = TRUE, append = FALSE)

write.csv(allCrosTabs,file = paste0(outputDir,'baseModelCompRSvsReported.csv'))

cat(allSumm,file = paste0(outputDir,'baseModelCompRSvsReported.txt'))

##----- 1.3. base model controlling for self assess as well as the four themes of SVI ----

covariates<- c('Male','AgeGrp', 'RaceGroup', 'EducGroup',"SelfAssess","reRankSVI_T1","reRankSVI_T2", "reRankSVI_T3", "reRankSVI_T4") #,'Hispanic'

outcomes<- c("Illness", "Injury","Hospital", "Concentrate", "Headaches", "RunnyNose", "ShortBreath", "SkinRash","AnySymptoms")

exposures<-c("OtherHomesFlooded","HomeFlooded","fScanFlooded","fScanInunDisCat","fScanNdaysCat" ,"fScanDepthCat","fScanDepthFt","fScanInundDist","fScanNdays")

print(paste0(c('outcomes-> ',outcomes),collapse = ', '))

print(paste0(c('exposures-> ',exposures),collapse = ', '))

print(paste0(c('covariates-> ',covariates),collapse = ', '))

summary(subDf[,c(covariates,exposures,outcomes)])

outcome<-outcomes[2]

exposure<- exposures[1]

allRes<-NA

allSumm<-''

allCrosTabs<-data.frame()

for(exposure in exposures)

for(outcome in outcomes){

mformula<-paste0(outcome,' ~ ', exposure, ' + ' , paste0(covariates,collapse = ' + '))

df<-subDf[complete.cases(subDf[,c(outcome,exposure,covariates,'tractID10')]),]

df['id']<-seq(dim(df)[1])

model<-geeglm(as.formula(mformula),

id=tractID10,

data = df,

family =poisson(link='log'),

corstr = "independence")

#update correlation structure

model2 <- update(model, corstr = "exch")

model3 <- update(model, corstr = "ar1")

##select correlation structure based on smallest QIC

modelSel<-list(model,model2,model3)[which.min(unlist(lapply(list(model,model2,model3),mQIC)))][[1]]

remove(model,model2,model3)

##get summary from the selected model

qic<- QIC(modelSel)

#summary(modelSel)

#broom results

resTab<-tidy(modelSel, conf.int = TRUE,exponentiate = T)

resTab[,c('estimate','p.value','conf.low','conf.high')]<-round(resTab[,c('estimate','p.value','conf.low','conf.high')],3)

resTab<-resTab[,c("term", "estimate","conf.low", "conf.high","p.value", "std.error", "statistic")]

#insert exposure,outcome and formula and Qic

resTab$qic<-qic['QIC']

resTab$outcome<-outcome

resTab$exposure<-exposure

resTab$formula<-mformula

#correlation struture

resTab$corstr<-modelSel$corstr

resTab$Nrow<-nrow(modelSel$data)

resTab$id<-as.character(modelSel$call$id)

resTab$otherCom<-""

#print(resTab)

#prepare raw summary file

sumTxt<-paste0(c(capture.output(summary(modelSel)),

'\n QIC:',capture.output(qic),

'Time:',as.character(Sys.time()),'\n'),collapse = '\n')

#replace formula with orginal formula

sumTxt<-gsub('mformula',mformula,sumTxt)

#add to all output stored df and str

if(is.na(allRes)) allRes<-resTab else allRes<-rbind(allRes,resTab)

allSumm<-paste0(allSumm,sumTxt,sep=paste0(rep('=',100),collapse=''))

print(mformula)

#create cross table if intersection columns are not factor

if(is.factor(df[,exposure]) | is.logical(df[,exposure])){

ftab<-data.frame(ftable(df[,outcome],df[,exposure],dnn=c(outcome,exposure)),stringsAsFactors = F)

ftab$formula<-mformula

ftab<-rbind(colnames(ftab),sapply(ftab,as.character))

colnames(ftab) <- as.character(seq(dim(ftab)[2]))

allCrosTabs<-rbind(allCrosTabs,ftab)

}

}

#write output files

allRes$model<-'baseModelCntrlSVI'

write.xlsx(allRes, file=paste0(outputDir,'baseModelCntrlSVI.xlsx'), sheetName = "Sheet1",col.names = TRUE, row.names = TRUE, append = FALSE)

write.csv(allCrosTabs,file = paste0(outputDir,'baseModelCntrlSVI.csv'))

cat(allSumm,file = paste0(outputDir,'baseModelCntrlSVI.txt'))

##----- 1.4. base model with overall svi cat as a covariate ----

covariates<- c("cat_SVI",'Male','AgeGrp', 'RaceGroup', 'EducGroup',"SelfAssess") #,'Hispanic'

outcomes<- c("Illness", "Injury","Hospital", "Concentrate", "Headaches", "RunnyNose", "ShortBreath", "SkinRash","AnySymptoms")

exposures<-c("OtherHomesFlooded","HomeFlooded","fScanFlooded","fScanInunDisCat","fScanNdaysCat" ,"fScanDepthCat","fScanDepthFt","fScanInundDist","fScanNdays")

print(paste0(c('outcomes-> ',outcomes),collapse = ', '))

print(paste0(c('exposures-> ',exposures),collapse = ', '))

print(paste0(c('covariates-> ',covariates),collapse = ', '))

summary(subDf[,c(covariates,exposures,outcomes)])

outcome<-outcomes[2]

exposure<- exposures[1]

allRes<-NA

allSumm<-''

allCrosTabs<-data.frame()

for(exposure in exposures)

for(outcome in outcomes){

mformula<-paste0(outcome,' ~ ', exposure, ' + ' , paste0(covariates,collapse = ' + '))

df<-subDf[complete.cases(subDf[,c(outcome,exposure,covariates,'tractID10','resp_id')]),]

df['id']<-seq(dim(df)[1])

model<-geeglm(as.formula(mformula),

id=tractID10,

data = df,

family =poisson(link='log'),

corstr = "independence")

#update correlation structure

model2 <- update(model, corstr = "exch")

model3 <- update(model, corstr = "ar1")

##select correlation structure based on smallest QIC

modelSel<-list(model,model2,model3)[which.min(unlist(lapply(list(model,model2,model3),mQIC)))][[1]]

remove(model,model2,model3)

##get summary from the selected model

qic<- QIC(modelSel)

#summary(modelSel)

print(mformula)

#run morans I

hrr_nbSub <- subset(hhr_nb, subDf$resp_id %in% df$resp_id)

moran_res <- moran.test(resid(modelSel), nb2listw(hrr_nbSub, style="W"))

print(moran_res)

#broom results

resTab<-tidy(modelSel, conf.int = TRUE,exponentiate = T)

resTab[,c('estimate','p.value','conf.low','conf.high')]<-round(resTab[,c('estimate','p.value','conf.low','conf.high')],3)

resTab<-resTab[,c("term", "estimate","conf.low", "conf.high","p.value", "std.error", "statistic")]

#insert exposure,outcome and formula and Qic

resTab$qic<-qic['QIC']

resTab$outcome<-outcome

resTab$exposure<-exposure

resTab$formula<-mformula

#correlation struture

resTab$corstr<-modelSel$corstr

resTab$Nrow<-nrow(modelSel$data)

resTab$id<-as.character(modelSel$call$id)

resTab$otherCom<-""

#print(resTab)

#prepare raw summary file

sumTxt<-paste0(c(capture.output(summary(modelSel)),

'\n QIC:',capture.output(qic),

'Time:',as.character(Sys.time()),'\n'),collapse = '\n')

#replace formula with orginal formula

sumTxt<-gsub('mformula',mformula,sumTxt)

#add to all output stored df and str

if(is.na(allRes)) allRes<-resTab else allRes<-rbind(allRes,resTab)

allSumm<-paste0(allSumm,sumTxt,sep=paste0(rep('=',100),collapse=''))

#create cross table if intersection columns are not factor

if(is.factor(df[,exposure]) | is.logical(df[,exposure])){

ftab<-data.frame(ftable(df[,outcome],df[,exposure],dnn=c(outcome,exposure)),stringsAsFactors = F)

ftab$formula<-mformula

ftab<-rbind(colnames(ftab),sapply(ftab,as.character))

colnames(ftab) <- as.character(seq(dim(ftab)[2]))

allCrosTabs<-rbind(allCrosTabs,ftab)

}

}

#write output files

allRes$model<-'baseModelWithSVIcat'

write.xlsx(allRes, file=paste0(outputDir,'baseModelWithSVIcat.xlsx'), sheetName = "Sheet1",col.names = TRUE, row.names = TRUE, append = FALSE)

write.csv(allCrosTabs,file = paste0(outputDir,'baseModelWithSVIcat.csv'))

cat(allSumm,file = paste0(outputDir,'baseModelWithSVIcat.txt'))

##----- 2.base model not controlling for self assess ----

covariates<- c('Male','AgeGrp', 'RaceGroup', 'EducGroup') #,'Hispanic'

outcomes<- c("Illness", "Injury","Hospital", "Concentrate", "Headaches", "RunnyNose", "ShortBreath", "SkinRash","AnySymptoms")

exposures<-c("OtherHomesFlooded","HomeFlooded","fScanFlooded","fScanInunDisCat","fScanNdaysCat" ,"fScanDepthCat","fScanDepthFt","fScanInundDist","fScanNdays")

print(paste0(c('outcomes-> ',outcomes),collapse = ', '))

print(paste0(c('exposures-> ',exposures),collapse = ', '))

print(paste0(c('covariates-> ',covariates),collapse = ', '))

summary(subDf[,c(covariates,exposures,outcomes)])

allRes<-NA

allSumm<-''

allCrosTabs<-data.frame()

for(exposure in exposures)

for(outcome in outcomes){

mformula<-paste0(outcome,' ~ ', exposure, ' + ' , paste0(covariates,collapse = ' + '))

df<-subDf[complete.cases(subDf[,c(outcome,exposure,covariates,'tractID10')]),]

df['id']<-seq(dim(df)[1])

model<-geeglm(as.formula(mformula),

id=tractID10,

data = df,

family =poisson(link='log'),

corstr = "independence")

#update correlation structure

model2 <- update(model, corstr = "exch")

model3 <- update(model, corstr = "ar1")

##select correlation structure based on smallest QIC

modelSel<-list(model,model2,model3)[which.min(unlist(lapply(list(model,model2,model3),mQIC)))][[1]]

remove(model,model2,model3)

##get summary from the selected model

qic<- QIC(modelSel)

#summary(modelSel)

#broom results

resTab<-tidy(modelSel, conf.int = TRUE,exponentiate = T)

resTab[,c('estimate','p.value','conf.low','conf.high')]<-round(resTab[,c('estimate','p.value','conf.low','conf.high')],3)

resTab<-resTab[,c("term", "estimate","conf.low", "conf.high","p.value", "std.error", "statistic")]

#insert exposure,outcome and formula and Qic

resTab$qic<-qic['QIC']

resTab$outcome<-outcome

resTab$exposure<-exposure

resTab$formula<-mformula

#correlation struture

resTab$corstr<-modelSel$corstr

resTab$Nrow<-nrow(modelSel$data)

resTab$id<-as.character(modelSel$call$id)

resTab$otherCom<-""

#print(resTab)

#prepare raw summary file

sumTxt<-paste0(c(capture.output(summary(modelSel)),

'\n QIC:',capture.output(qic),

'Time:',as.character(Sys.time()),'\n'),collapse = '\n')

#replace formula with orginal formula

sumTxt<-gsub('mformula',mformula,sumTxt)

#add to all output stored df and str

if(is.na(allRes)) allRes<-resTab else allRes<-rbind(allRes,resTab)

allSumm<-paste0(allSumm,sumTxt,sep=paste0(rep('=',100),collapse=''))

print(mformula)

#create cross table if intersection columns are not factor

if(is.factor(df[,exposure]) | is.logical(df[,exposure])){

ftab<-data.frame(ftable(df[,outcome],df[,exposure],dnn=c(outcome,exposure)),stringsAsFactors = F)

ftab$formula<-mformula

ftab<-rbind(colnames(ftab),sapply(ftab,as.character))

colnames(ftab) <- as.character(seq(dim(ftab)[2]))

allCrosTabs<-rbind(allCrosTabs,ftab)

}

}

allRes$model<-'baseModelNotAdjSelfAssess'

#write output files

write.xlsx(allRes, file=paste0(outputDir,'baseModelNotCntrlSelfAsses.xlsx'), sheetName = "Sheet1",col.names = TRUE, row.names = TRUE, append = FALSE)

write.csv(allCrosTabs,file = paste0(outputDir,'baseModelNotCntrlSelfAsses.csv'))

cat(allSumm,file = paste0(outputDir,'baseModelNotCntrlSelfAsses.txt'))

##----- 3.model not controlling for any covariates ----

covariates<- c('Male','AgeGrp', 'RaceGroup', 'EducGroup',"SelfAssess") #,'Hispanic'

outcomes<- c("Illness", "Injury","Hospital", "Concentrate", "Headaches", "RunnyNose", "ShortBreath", "SkinRash","AnySymptoms")

exposures<-c("OtherHomesFlooded","HomeFlooded","fScanFlooded","fScanInunDisCat","fScanNdaysCat" ,"fScanDepthCat")

print(paste0(c('outcomes-> ',outcomes),collapse = ', '))

print(paste0(c('exposures-> ',exposures),collapse = ', '))

summary(subDf[,c(exposures,outcomes)])

allRes<-NA

allSumm<-''

allCrosTabs<-data.frame()

for(exposure in exposures)

for(outcome in outcomes){

mformula<-paste0(outcome,' ~ ', exposure)

df<-subDf[complete.cases(subDf[,c(outcome,exposure,'tractID10')]),]

df['id']<-seq(dim(df)[1])

model<-geeglm(as.formula(mformula),

id=tractID10,

data = df,

family =poisson(link='log'),

corstr = "independence")

#update correlation structure

model2 <- update(model, corstr = "exch")

model3 <- update(model, corstr = "ar1")

##select correlation structure based on smallest QIC

modelSel<-list(model,model2,model3)[which.min(unlist(lapply(list(model,model2,model3),mQIC)))][[1]]

remove(model,model2,model3)

##get summary from the selected model

qic<- QIC(modelSel)

#summary(modelSel)

#broom results

resTab<-tidy(modelSel, conf.int = TRUE,exponentiate = T)

resTab[,c('estimate','p.value','conf.low','conf.high')]<-round(resTab[,c('estimate','p.value','conf.low','conf.high')],3)

resTab<-resTab[,c("term", "estimate","conf.low", "conf.high","p.value", "std.error", "statistic")]

#insert exposure,outcome and formula and Qic

resTab$qic<-qic['QIC']

resTab$outcome<-outcome

resTab$exposure<-exposure

resTab$formula<-mformula

#correlation struture

resTab$corstr<-modelSel$corstr

resTab$Nrow<-nrow(modelSel$data)

resTab$id<-as.character(modelSel$call$id)

resTab$otherCom<-""

#print(resTab)

#prepare raw summary file

sumTxt<-paste0(c(capture.output(summary(modelSel)),

'\n QIC:',capture.output(qic),

'Time:',as.character(Sys.time()),'\n'),collapse = '\n')

#replace formula with orginal formula

sumTxt<-gsub('mformula',mformula,sumTxt)

#add to all output stored df and str

if(is.na(allRes)) allRes<-resTab else allRes<-rbind(allRes,resTab)

allSumm<-paste0(allSumm,sumTxt,sep=paste0(rep('=',100),collapse=''))

print(mformula)

#create cross table if intersection columns are not factor

if(is.factor(df[,exposure]) | is.logical(df[,exposure])){

ftab<-data.frame(ftable(df[,outcome],df[,exposure],dnn=c(outcome,exposure)),stringsAsFactors = F)

ftab$formula<-mformula

ftab<-rbind(colnames(ftab),sapply(ftab,as.character))

colnames(ftab) <- as.character(seq(dim(ftab)[2]))

allCrosTabs<-rbind(allCrosTabs,ftab)

}

}

#write output files

allRes$model<-'baseModelNotAdjAnyCovariates'

write.xlsx(allRes, file=paste0(outputDir,'baseModelNotCntrlAnything.xlsx'), sheetName = "Sheet1",col.names = TRUE, row.names = TRUE, append = FALSE)

write.csv(allCrosTabs,file = paste0(outputDir,'baseModelNotCntrlAnything.csv'))

cat(allSumm,file = paste0(outputDir,'baseModelNotCntrlAnything.txt'))

##------ 4.base model with interaction for individual level characteristic and controlling for self assess ------

inter='RaceGroup'

exposure='OnlyOtherHomesFlooded'

outcome='Illness'

covariates<- c('Male','AgeGrp', 'RaceGroup', 'EducGroup',"SelfAssess") #,'Hispanic'

outcomes<- c( "Concentrate")#, "Illness", "Injury","Hospital", "Headaches", "RunnyNose", "ShortBreath", "SkinRash","AnySymptoms")

exposures<-c("HomeFlooded","fScanFlooded")#"OnlyOtherHomesFlooded","OtherHomesFlooded","Contact_Water","fScanInunDisCat","fScanNdaysCat" ,"fScanDepthCat")

inters<-c('Male')#,'RaceGroup','AgeGrp', 'EducGroup')

print(paste0(c('outcomes-> ',outcomes),collapse = ', '))

print(paste0(c('exposures-> ',exposures),collapse = ', '))

print(paste0(c('covariates-> ',covariates),collapse = ', '))

summary(subDf[,c(covariates,exposures,outcomes,inters)])

allRes<-NA

allSumm<-''

allCrosTabs<-data.frame()

for(inter in inters){

for(exposure in exposures)

for(outcome in outcomes){

mformula<-paste0(outcome,' ~ ', exposure,' * ',inter,' + ' , paste0(covariates,collapse = ' + '))

df<-subDf[complete.cases(subDf[,c(outcome,exposure,covariates,inter,'tractID10')]),]

#combine race - asian and others

df$RaceGroup<-recode(df$RaceGroup,"c('Non_Hispanic_Asian', 'Non_Hispanic_Other')='Non_Hispanic_AsianNOthers'")

df <- df %>% mutate(RaceGroup=relevel(RaceGroup,ref='Non_Hispanic_White'))

#create cross table if intersection columns are not factor

if (is.factor(df[,inter])){

ftab<-data.frame(ftable(df[,outcome],df[,exposure],df[,inter],dnn=c(outcome,exposure,inter)),stringsAsFactors = F)

ftab$formula<-mformula

ftab1<-rbind(colnames(ftab),sapply(ftab,as.character))

colnames(ftab1) <- as.character(seq(dim(ftab1)[2]))

allCrosTabs<-rbind(allCrosTabs,ftab1)

#if any of the counts is 0 skip this loop

if(any(ftab$Freq==0)){

allSumm<-paste0(allSumm,'\n',mformula,'\n skipped due to not enought records count in each category bin \n',sep=paste0(rep('=',100),collapse=''))

next()}

}

df['id']<-seq(dim(df)[1])

model<-geeglm(as.formula(mformula),

id=tractID10,

data = df,

family =poisson(link='log'),

corstr = "independence")

#update correlation structure

model2 <- update(model, corstr = "exch")

model3 <- update(model, corstr = "ar1")

##select correlation structure based on smallest QIC

modelSel<-list(model,model2,model3)[which.min(unlist(lapply(list(model,model2,model3),mQIC)))][[1]]

remove(model,model2,model3)

##get summary from the selected model

qic<- QIC(modelSel)

#summary(modelSel)

#broom results

resTab<-tidy(modelSel, conf.int = TRUE,exponentiate = T)

resTab[,c('estimate','p.value','conf.low','conf.high')]<-round(resTab[,c('estimate','p.value','conf.low','conf.high')],3)

resTab<-resTab[,c("term", "estimate","conf.low", "conf.high","p.value", "std.error", "statistic")]

#insert exposure,outcome and formula and Qic

resTab$qic<-qic['QIC']

resTab$outcome<-outcome

resTab$exposure<-exposure

resTab$formula<-mformula

#correlation struture

resTab$corstr<-modelSel$corstr

resTab$Nrow<-nrow(modelSel$data)

resTab$id<-as.character(modelSel$call$id)

resTab$otherCom<-paste0('interaction term ',inter)

#print(resTab)

#prepare raw summary file

sumTxt<-paste0(c(capture.output(summary(modelSel)),

'\n QIC:',capture.output(qic),

'Time:',as.character(Sys.time()),'\n'),collapse = '\n')

#replace formula with orginal formula

sumTxt<-gsub('mformula',mformula,sumTxt)

#add to all output stored df and str

if(is.na(allRes)) allRes<-resTab else allRes<-rbind(allRes,resTab)

allSumm<-paste0(allSumm,sumTxt,sep=paste0(rep('=',100),collapse=''))

#print(ftab)

print(mformula)

}

cat(allSumm,file = paste0(outputDir,'demogInteractionModel.txt'),append = T)

allSumm<-''

}

allRes$model<-'demogInteractionModel'

#write output files

write.xlsx(allRes, file=paste0(outputDir,'demogInteractionModel.xlsx'), sheetName = "Sheet1",col.names = TRUE, row.names = TRUE, append = FALSE)

write.csv(allCrosTabs,file = paste0(outputDir,'demogInteractionModel.csv'))

##------ 5.base model with SVI interaction and controlling for self assess ------

covariates<- c('Male','AgeGrp', 'RaceGroup', 'EducGroup',"SelfAssess") #,'Hispanic'

outcomes<- c("Illness", "Injury","Hospital", "Concentrate", "Headaches", "RunnyNose", "ShortBreath", "SkinRash","AnySymptoms")

exposures<-c("OtherHomesFlooded","HomeFlooded","fScanFlooded","fScanInunDisCat","fScanNdaysCat" ,"fScanDepthCat")

inters<-c("reRankSVI", "reRankSVI_T1", "reRankSVI_T2", "reRankSVI_T3", "reRankSVI_T4",

"cat_SVI", "cat_SVI_T1", "cat_SVI_T2", "cat_SVI_T3", "cat_SVI_T4")

print(paste0(c('outcomes-> ',outcomes),collapse = ', '))

print(paste0(c('exposures-> ',exposures),collapse = ', '))

print(paste0(c('covariates-> ',covariates),collapse = ', '))

summary(subDf[,c(covariates,exposures,outcomes,inters)])

outcome<-outcomes[2]

exposure<- exposures[1]

allRes<-NA

allSumm<-''

allCrosTabs<-data.frame()

for(inter in inters){

for(exposure in exposures)

for(outcome in outcomes){

mformula<-paste0(outcome,' ~ ', exposure,' * ',inter,' + ' , paste0(covariates,collapse = ' + '))

df<-subDf[complete.cases(subDf[,c(outcome,exposure,covariates,inter,'tractID10')]),]

#create cross table if intersection columns are not factor

if (is.factor(df[,inter])){

ftab<-data.frame(ftable(df[,outcome],df[,exposure],df[,inter],dnn=c(outcome,exposure,inter)),stringsAsFactors = F)

ftab$formula<-mformula

ftab1<-rbind(colnames(ftab),sapply(ftab,as.character))

colnames(ftab1) <- as.character(seq(dim(ftab1)[2]))

allCrosTabs<-rbind(allCrosTabs,ftab1)

#if any of the counts is 0 skip this loop

if(any(ftab$Freq==0)){

allSumm<-paste0(allSumm,'\n',mformula,'\n skipped due to not enought records count in each category bin \n',sep=paste0(rep('=',100),collapse=''))

next()}

}

df['id']<-seq(dim(df)[1])

model<-geeglm(as.formula(mformula),

id=tractID10,

data = df,

family =poisson(link='log'),

corstr = "independence")

#update correlation structure

model2 <- update(model, corstr = "exch")

model3 <- update(model, corstr = "ar1")

##select correlation structure based on smallest QIC

modelSel<-list(model,model2,model3)[which.min(unlist(lapply(list(model,model2,model3),mQIC)))][[1]]

remove(model,model2,model3)

##get summary from the selected model

qic<- QIC(modelSel)

#summary(modelSel)

#broom results

resTab<-tidy(modelSel, conf.int = TRUE,exponentiate = T)

resTab[,c('estimate','p.value','conf.low','conf.high')]<-round(resTab[,c('estimate','p.value','conf.low','conf.high')],3)

resTab<-resTab[,c("term", "estimate","conf.low", "conf.high","p.value", "std.error", "statistic")]

#insert exposure,outcome and formula and Qic

resTab$qic<-qic['QIC']

resTab$outcome<-outcome

resTab$exposure<-exposure

resTab$formula<-mformula

#correlation struture

resTab$corstr<-modelSel$corstr

resTab$Nrow<-nrow(modelSel$data)

resTab$id<-as.character(modelSel$call$id)

resTab$otherCom<-paste0('interaction term ',inter)

#print(resTab)

#prepare raw summary file

sumTxt<-paste0(c(capture.output(summary(modelSel)),

'\n QIC:',capture.output(qic),

'Time:',as.character(Sys.time()),'\n'),collapse = '\n')

#replace formula with orginal formula

sumTxt<-gsub('mformula',mformula,sumTxt)

#add to all output stored df and str

if(is.na(allRes)) allRes<-resTab else allRes<-rbind(allRes,resTab)

allSumm<-paste0(allSumm,sumTxt,sep=paste0(rep('=',100),collapse=''))

#print(ftab)

print(mformula)

}

#write some outputs to clear memorty

cat(allSumm,file = paste0(outputDir,'sviInteractionModel.txt'),append = T)

allSumm<-''

write.table(allCrosTabs, file = paste0(outputDir,'sviInteractionModel.csv'), sep = ",",

col.names = !file.exists(paste0(outputDir,'sviInteractionModel.csv')), append = T)

allCrosTabs<-data.frame()

}

allRes$model<-'sviInteractionModel'

#write output files

write.xlsx(allRes, file=paste0(outputDir,'sviInteractionModel.xlsx'), sheetName = "Sheet1",col.names = TRUE, row.names = TRUE, append = FALSE)
